# Development of Granzyme A Turn‐ON Fluorescent Activity‐Based Probes

**DOI:** 10.1002/cbic.202500771

**Published:** 2025-11-19

**Authors:** Sebastian M. Malespini, Muhammad Kazim, Euna Yoo

**Affiliations:** ^1^ Chemical Biology Laboratory Center for Cancer Research National Cancer Institute National Institutes of Health Frederick MD 21702 USA

**Keywords:** activity‐based probes, granzyme A, immunity, optical imaging, protease

## Abstract

Granzyme A (GzmA), a serine protease highly expressed in cytotoxic immune cells, plays complex roles in antitumor immunity and inflammation. While elevated GzmA levels correlate with favorable outcomes in certain cancers, extracellular GzmA has been implicated in promoting tumorigenesis via inflammatory pathways. These contrasting functions highlight the need for selective tools that can detect GzmA activity with high spatiotemporal resolution in native biological contexts. A series of quenched activity‐based probes that fluoresce only upon covalent binding to active GzmA have been developed. The lead probe, featuring the Ile–Gly–Asn–Arg recognition sequence, a phenyl phosphinate ester warhead, and a near‐infrared sulfo‐Cy5/QSY21 fluorophore/quencher pair, exhibits high reactivity, selectivity, and cell permeability. It enables robust detection of GzmA in vitro and in cells, with an excellent signal‐to‐noise ratio. These activatable probes will support downstream activity‐based protein profiling and enable noninvasive imaging of the enzyme activity, offering a powerful means for dissecting the multifaceted biology of GzmA within the tumor immune microenvironment.

## Graphical abstract

A quenched activity‐based probe, featuring the Ile–Gly–Asn–Arg recognition sequence, a phenyl phosphinate ester warhead, and a near‐infrared sulfo‐Cy5/QSY21 fluorophore/quencher pair, generates a fluorescence signal only upon reaction with active granzyme A (GzmA). With its high reactivity, selectivity, and cell permeability, this turn‐ON probe enables a robust detection of GzmA activity in complex proteomes.

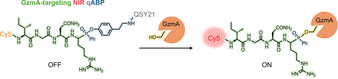

## 1 Introduction

Granzymes (Gzms) are a family of serine proteases (A, B, H, K, and M) that are stored in the cytolytic granules of natural killer (NK) cells and cytotoxic T lymphocytes. Upon target cell recognition, degranulation is triggered, leading to the simultaneous release of Gzm and perforin into the immunological synapse. Perforin polymerizes to form transmembrane pores in the target cell, facilitating Gzm entry into the cytosol and initiating cell death through diverse mechanisms.^[^ ^1^ ^]^ Granzyme A (GzmA), the most abundant granzyme in humans, has been shown to induce caspase‐independent cell death resembling apoptosis,^[^ ^1^ ^]^ though pyroptosis may also occur in cells expressing gasdermin B.^[^ ^2^ ^]^ In breast cancer, elevated levels of GzmA within the tumor immune microenvironment (TIME) correlate with improved disease‐specific survival and progression‐free interval. GzmA expression is also positively associated with increased infiltration of dendritic and CD8+ T cells, suggesting its cytotoxic function in antitumor immunity.^[^ ^3^ ^]^ Paradoxically, extracellular GzmA has been implicated in promoting colorectal cancer through macrophage‐mediated release of interleukin (IL)‐6, a proinflammatory cytokine that activates STAT3‐driven tumorigenesis.^[^ ^4^ ^]^ These multifaceted roles underscore the need for selective tools to detect and study GzmA in a cell and context‐specific manner with high spatiotemporal resolution.

Activity‐based probes (ABPs) offer a powerful means to directly monitor enzyme activity in complex biological systems. ABPs typically comprise three key elements: a peptide recognition sequence conferring enzyme specificity, a reactive warhead that forms an irreversible covalent bond with the active site catalytic residue, and a reporter tag, often a fluorophore or affinity handle, for detection or enrichment. ABPs have been employed in activity‐based protein profiling (ABPP) platforms, such as in‐gel fluorescence assays, mass spectrometry (MS)‐based proteomics, and molecular imaging, to assess enzyme activity and functional states.^[^ ^5^ ^]^ For example, a GzmA‐specific ABP incorporating a Tic–Gly–Oic–Arg tetrapeptide, a diphenyl phosphonate warhead, and a Cy5 fluorophore enabled detection of active GzmA in NK cells via in‐gel fluorescence analysis.^[^ ^6^ ^]^ However, the always‐ON nature of ABPs leads to high background signals, necessitating extensive washing steps for the removal of unreacted probes, which challenges real‐time enzymatic activity monitoring in complex settings.^[^ ^7^ ^]^

On the other hand, chemical strategies to generate the GzmA turn‐ON signal have primarily focused on fluorogenic substrate‐based probes. The Vendrell group, for example, designed probes linking GzmA substrate peptides to the hemicyanine near‐infrared (NIR) fluorophore via a self‐immolating *p*‐aminobenzyl alcohol (PABA) linker, enabling signal generation exclusively upon enzymatic cleavage.^[^ ^8^ ^]^ FRET‐based probes featuring a Dabcyl quencher and a FAM fluorophore have also been reported to detect extracellular GzmA activity.^[^ ^9^ ^]^ These probes are inherently quenched but activated by GzmA cleavage, allowing for an enhanced signal‐to‐noise (S/N) ratio with minimal background. However, the specificity of such probes must be extensively characterized since the responsible enzyme(s) generating a signal cannot be directly identified in complex environments. Additionally, probe diffusion after being cleaved may prevent significant signal accumulation and contrast at the site of target engagement.^[^ ^10^ ^]^

To address these limitations, we developed a series of quenched ABPs (qABPs) that produce a fluorescence signal only after covalent binding to active GzmA and remain tethered to the enzyme. We utilized a phenyl phosphinate warhead, which enables efficient quencher release upon conjugation while preserving the reactivity of classical diphenyl phosphonates with the catalytic serine residue. For optimal imaging potential, we incorporated the NIR fluorophore sulfonated‐Cy5 into GzmA‐selective tetrapeptide sequences, capitalizing on its deep tissue penetration, low scattering, and minimal overlap with tissue autofluorescence.^[^ ^11^ ^,^ ^12^ ^]^ The resulting qABPs exhibited excellent activation kinetics and selectivity for GzmA in both in vitro and cellular contexts, achieving high S/N ratios. Thus, these probes offer a valuable chemical toolkit for elucidating the complex roles that GzmA plays in the TIME.

## 2 Results and Discussion

**Figure 1:**
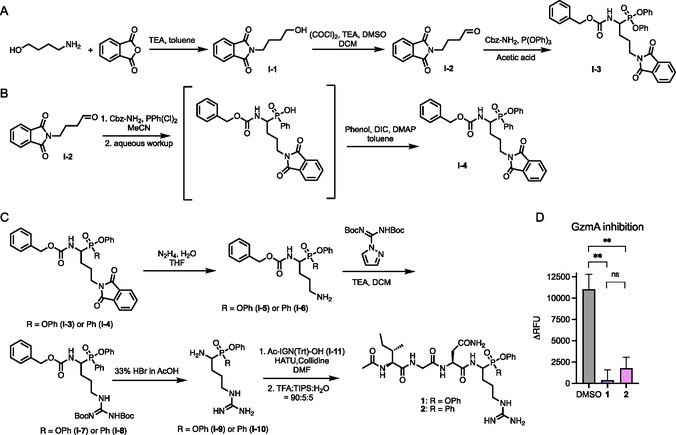
GzmA phosphonate and phosphinate inhibitors. A) Synthesis of phosphonate warhead **I‐3**. B) Synthesis of phosphosphinate warhead **I‐4**. C) Synthesis of Ac‐IGNR‐PO(OPh)_2_ **1** and Ac‐IGNR‐PO(OPh)Ph **2**. D) GzmA inhibition. hGzmA (50 nM) was incubated with inhibitor **1** or **2** (200 nM) and GzmA fluorogenic substrate (Ac‐Oic‐Gly‐Pro‐Arg‐PABA‐MU, 100 μM) for 15 h at 37 °C. Fluorescence at Ex/Em: 380/460 nm was recorded for residual GzmA proteolytic activity. Data points are displayed as mean ± SD (*n* = 3), and the *p*‐value was evaluated by the Student's *t*‐test (**p‐value ≤ 0.01).

Diphenyl phosphonate warhead has been widely used to target serine proteases due to its high reactivity and selectivity.^[^ ^6^ ^,^ ^13^ ^,^ ^14^ ^]^ However, its use in activatable probe design is limited by the presence of competing phenol leaving groups. Exploiting the differences in charge stability between alkoxy and phenoxy groups, others have designed mixed phosphonates as activatable ABPs.^[^ ^15^, ^16^, ^17^ ^]^ Yet, these warheads have not gained widespread utility, primarily due to the synthetic challenges associated with their design and hydrolytic instability. We recently reported a qABP featuring a phosphinate ester warhead^[^ ^18^ ^]^ for detecting GzmB activity in tumors undergoing immunotherapy.^[^ ^19^ ^]^ Building on this, we sought to develop analogous probes for GzmA. To evaluate the suitability of a phosphinate warhead for GzmA‐targeting qABPs, we first synthesized phosphonate and phosphinate inhibitors **1** and **2**, respectively, each incorporating the GzmA substrate sequence Ile–Gly–Asn–Arg (IGNR)^[^ ^20^ ^]^ (**Figure** **1**). Briefly, the protection of phthalic anhydride with 4‐aminobutanol followed by Swern oxidation to yield aldehyde intermediate **I‐2** (Figure 1A). A three‐component reaction with benzyl carbamate and either triphenyl phosphite or dichlorophenyl phosphine produced the corresponding phosphonate (**I‐3**, Figure 1A) and phosphinate (**I‐4**, Figure 1B) intermediates, respectively. Deprotection of the phtalimide group yielded amines **I‐5** and **I‐6**, which were subsequently reacted with bis‐Boc‐pyrazolocarboxamidine to generate intermediates **I‐7** and **I‐8** (Figure 1C). Global deprotection of Cbz and Boc groups with strong acid afforded the phosphonate and phosphinate cores (**I‐9** and **I**‐**10**), which were then coupled to the tripeptide Ac‐IGN(Trt)‐OH (**I‐11**). Subsequent deprotection with TFA yielded final inhibitors **1** and **2**. Both compounds were found to inhibit GzmA‐mediated cleavage of a fluorogenic substrate with comparable potency, confirming that the phosphinate warhead is a viable alternative to phosphonates for incorporation into GzmA‐targeting ABPs (Figure 1D).

**Figure 2:**
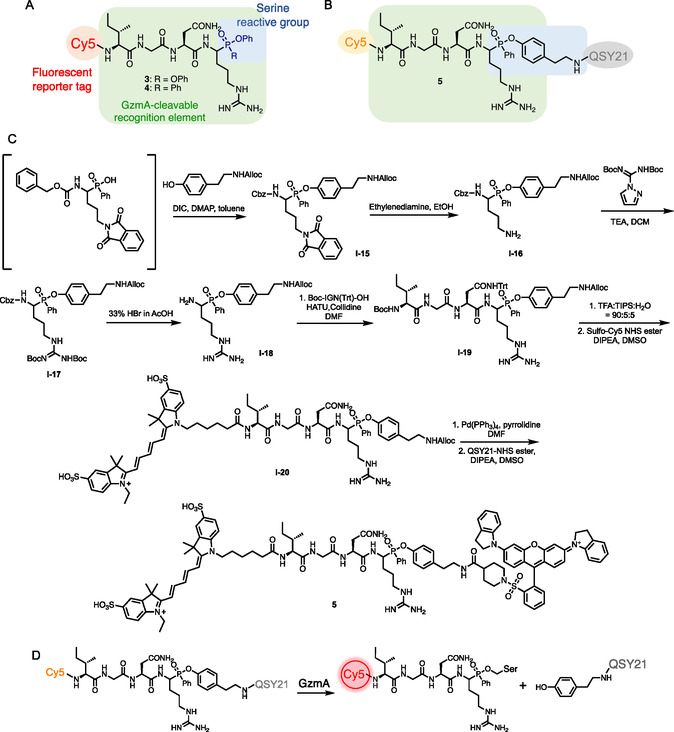
Design and synthesis of gzmA qABP. A) Chemical structures of phosphonate and phosphinate ABPs **3** and **4**, respectively. B) Chemical structure of GzmA qABP **5**. C) Synthesis of qABP **5**. D) Covalent reaction mechanism of qABP **5**.

We next synthesized fluorescent ABPs **3** and **4** (**Figure** **2**A and Scheme S1, Supporting Information) and a first‐generation qABP **5** for GzmA (Figure 2B,C). Probe **5** was prepared by esterifying the phosphinic acid intermediate with alloc‐tyramine to generate intermediate **I‐15.** For the removal of phthalimide protecting group, we used ethylenediamine instead of hydrazine, as the latter was found to reduce the alloc‐double bond. Subsequent guanidinylation, deprotection of the Boc and Cbz groups, and coupling to the tripeptide and sulfo‐Cy5 produced intermediate **I‐20**. The alloc group was removed using catalytic Pd(PPh_3_)_4_ and pyrrolidine, and the resulting amine was treated with QSY21 NHS ester to afford the final qABP **5**.

**Figure 3:**
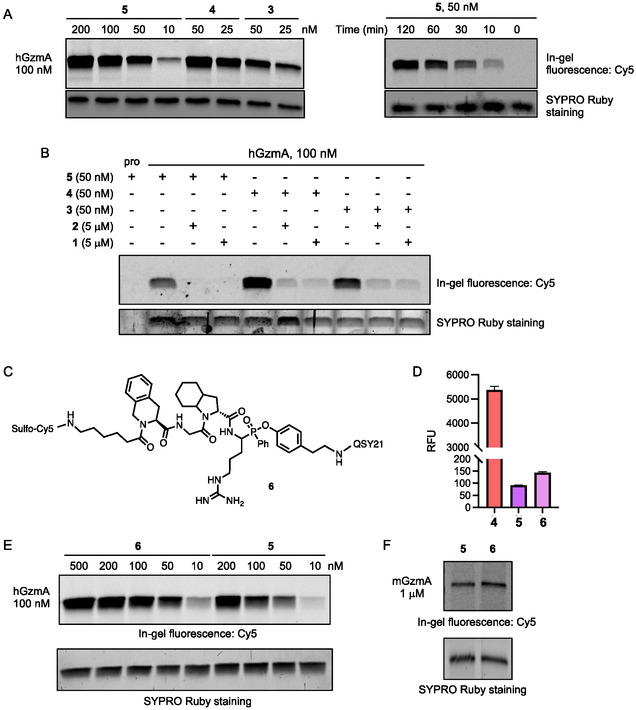
GzmA ABPs covalently and competitively label active human and mouse GzmA. A) Concentration‐ and time‐dependent labeling of hGzmA with **3–5**. Recombinant hGzmA (100 nM) was incubated with varying concentrations of each ABP at 37 °C. B) Competitive labeling of hGzmA. Recombinant hGzmA (100 nM) was preincubated with covalent inhibitors **1** or **2** (5 μM) for 2 h, followed by labeling with ABPs **3** **–5** (50 nM) for 2 h at 37 °C. Note: Pro‐form of hGzmA (the first lane) is not effectively stained by SYPRO‐Ruby. C) Structure of qABP **6**. D) Quenched natures of **5** and **6**. Data points are displayed as mean ± SD (*n* = 3) E) Concentration‐dependent labeling of hGzmA with **6** in comparison with **5** (2 h, 37 °C). F) Labeling of mGzmA (1 μM) with **5** and **6** (1 μM, 24 h, 37 °C). Protein samples were analyzed by SDS‐PAGE and in‐gel fluorescence scanning for Cy5 signal and SYPRO Ruby staining.

The NIR fluorescence signal in **5** was efficiently quenched (**Figure** **3**D) and expected to “turn‐ON” only upon covalent reaction with active GzmA, which separates the quencher from the fluorophore (Figure 2D). In vitro labeling studies confirmed that probes **3, 4**, and **5** efficiently label recombinant human GzmA (hGzmA) at submicromolar concentrations (Figure 3A). This labeling was competitively blocked by pretreatment with 5 μM of inhibitor **1** or **2** (Figure 3B). Importantly, qABP **5** showed concentration‐ and time‐dependent labeling of active hGzmA, with signal intensity comparable to that of probes **3** and **4** (Figure 3A). No labeling of the pro‐form of GzmA (without signal peptide) was observed (Figure 3B), highlighting the probe's selectivity for the catalytically active enzyme. Additionally, qABP **6** was synthesized using a recognition sequence previously reported for GzmA,^[^ ^6^ ^]^ featuring non‐natural amino acids L‐octahydroindole‐2‐carboxylic acid (Oic) at P2 and L‐1,2,3,4‐tetrahydroisoquinoline‐3‐carboxylic acid (Tic) at P4 positions (Figure 3C and Scheme S2, Supporting Information). Probe **6** efficiently (and slightly more strongly than **5**) labeled both human (Figure 3E) and mouse GzmA (mGzmA) (Figure 3F). We observed that even after prolonged incubation (24 h) and at higher probe concentrations (200 nM and 1 μM), the intensity of fluorescently labeled mGzmA (1 μM) is markedly weaker than that of hGzmA (Figure S1B,C, Supporting Information). To further enhance probe specificity toward mGzmA, we also synthesized qABP **7** (Figure S1A, Supporting Information), incorporating the GFFR recognition sequence previously reported to confer greater selectivity for mGzmA.^[^ ^21^ ^]^ Despite this modification, probe **7** exhibited lower labeling efficiency for both hGzmA and mGzmA compared with probes **5** and **6** (Figures S1B,C, Supporting Information). Because no ABPs for mGzmA have been reported to date, it remains unclear whether this weaker labeling arises from inherently lower enzymatic activity of mGzmA relative to hGzmA, or from reduced probe reactivity. Nonetheless, probe **6** showed concentration‐dependent labeling of mGzmA detected by in‐gel fluorescence (Figure S1D, Supporting Information). Further studies, including competitive labeling following inhibitor preincubation and determination of active enzyme concentration via substrate cleavage assays, are required to confirm activity‐dependent labeling of mGzmA by these probes.

To assess potential cross‐reactivity of our probes, we performed labeling assays with selected serine proteases, including neutrophil elastase (NE) and proteinase 3 (PR3) (Figure S2, Supporting Information). Neither probe **5** nor **6** showed detectable labeling of NE or PR3 (Figure S2A, Supporting Information), indicating minimal off‐target activity toward these proteases. Both GzmA and GzmK are granzyme tryptases that primarily cleave substrates after basic residues, such as Arg and Lys.^[^ ^22^ ^,^ ^23^ ^]^ Given their overlapping substrate specificities, we examined whether our qABPs could also label GzmK. Indeed, both probes **5** and **6** labeled GzmK (Figure S2B, Supporting Information); however, labeling was significantly weaker than for GzmA and required considerably higher probe concentrations (Figure S2C, Supporting Information). Interestingly, fluorescent ABPs **3** and **4** exhibited lower reactivity toward GzmK than qABPs **5** and **6**, suggesting distinct substrate engagement patterns at the primed subsites of GzmA and GzmK. These heretofore unknown results highlight subtle yet functionally relevant differences in probe‐enzyme interactions between these closely related granzymes, calling for further investigation. Although not examined in this study, additional potential off‐target proteins include cathepsin G (CatG), a serine protease found in neutrophils and stored in the azurophil granules.^[^ ^24^ ^]^ Given that CatG displays both chymotrypsin‐ and trypsin‐like specificities,^[^ ^25^ ^]^ it would be of interest to determine whether our probes also label active CatG.

**Figure 4:**
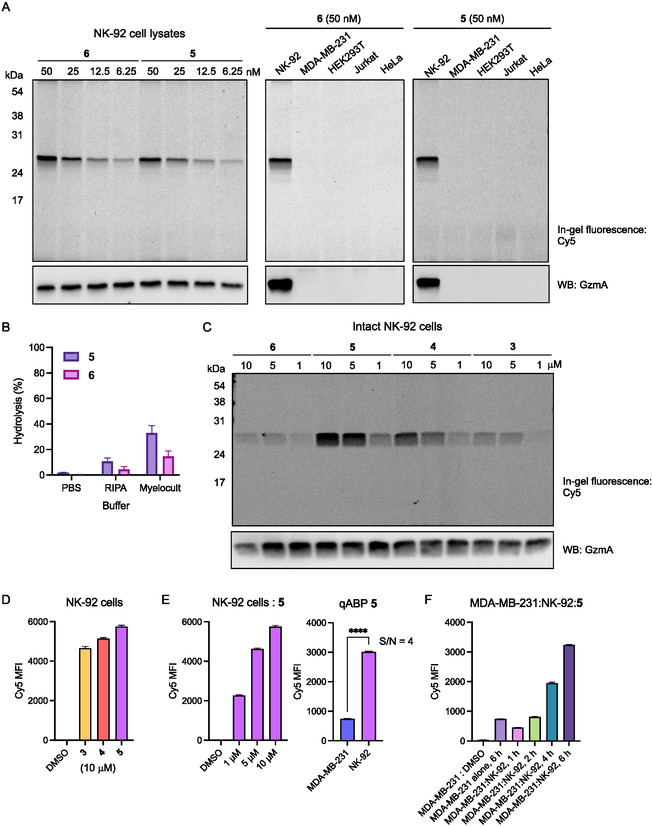
Characterization of qABPs in native proteomes. A) Labeling of GzmA by **5** and **6** in lysates of various cell lines (1 h, 37 °C). B) Stability of **5** and **6** in buffer and cell media (11 h, 37 °C). The hydrolysis of probes was measured by monitoring the production of Cy5 fluorescence signal over time. Data points are displayed as mean ± SD (*n* = 3). C) Labeling of GzmA by probes **3** **–6** in NK‐92 cells (2 h, 37 °C). D) Flow cytometry analysis of protein labeling in NK‐92 cells by **3** **–5**. Data points are displayed as mean ± SD (*n* = 3). E) Flow cytometry analysis of concentration‐dependent GzmA labeling in NK‐92 cells and S/N of **5** (5 μM, 2 h, 37 °C). Data points are displayed as mean ± SD (*n* = 3), and the *p*‐value was evaluated by the Student's t‐test (****p‐value ≤ 0.0001). F) Flow cytometry analysis of protein labeling by **5** in MDA‐MB‐231 cells co‐cultured with NK‐92 cells (see Supporting Information for conditions). Data points are displayed as mean ± SD (*n* = 3).

Next, we evaluated the performance of probes **5** and **6** in complex biochemical environments. In lysate of NK‐92 cells (immortalized human NK cell lines), both probes selectively labeled GzmA in a concentration‐dependent manner at low nanomolar levels, with no detectable cross‐reactivity in lysates of GzmA nonexpressing cells (MDA‐MB‐231, HEK293T, Jurkat, and HeLa cells) (**Figure** **4**A and Figure S3A, Supporting Information). Notably, incorporation of non‐natural amino acids in probe **6** was found to improve its stability, with only ≈20% hydrolysis observed after 11 h of incubation in cell culture media monitored by production of Cy5 fluorescence signal (Figure 4B). Despite its improved stability, probe **6** exhibited poor labeling of GzmA in intact NK‐92 cells, likely due to limited cell permeability (Figure 4C and Figure S3B, Supporting Information). In contrast, qABP **5** demonstrated excellent cellular uptake and effectively labeled active GzmA in intact cells. Flow cytometry analysis confirmed the cell permeability of probes **3** **–** **5**, with a clear Cy5 signal in NK‐92 cells (Figure 4D) and showed concentration‐dependent protein labeling by **5** (Figure 4E). Importantly, when comparing the mean fluorescence intensity between intact NK‐92 and MDA‐MB‐231 cells, probe **5** achieved a high S/N ratio of ≈4. This represents a substantial improvement over always‐ON ABP **4**, highlighting the advantage of quencher incorporation for enhancing signal contrast (Figure S4, Supporting Information). Furthermore, probe **5** was able to detect GzmA transfer from immune effector NK‐92 cells to target MDA‐MB‐231 cells in coculture conditions, as demonstrated by both flow cytometry and gel‐based analyses (Figure 4F and Figure S5, Supporting Information).

Collectively, these results demonstrate that qABP **5** is a robust and selective tool for detecting active GzmA in complex biological systems and can facilitate deeper investigations into the role of GzmA in the TIME.

## 3 Conclusion

Despite its implication in both immune‐mediated tumor cell killing and chronic inflammation, the role of GzmA in the TIME remains incompletely understood. This is due in part to the lack of chemical tools capable of selectively interrogating GzmA activity in complex biological systems. To this end, we have developed a novel GzmA qABP **5**, featuring the IGNR recognition sequence, a phenyl phosphinate warhead, and an NIR sulfo‐Cy5/QSY21 fluorophore/quencher pair. Probe **5** covalently labels active GzmA in a time‐ and concentration‐dependent manner and demonstrates high selectivity and cell permeability, as confirmed in both cell lysates and intact NK‐92 cells. Importantly, **5** exhibits a high S/N, highlighting the advantages of qABP for minimizing background noise and enhancing signal contrast in complex environments.

The biological utility of GzmA‐targeting ABPs extends to in vivo imaging applications, where they could serve as noninvasive tools to monitor GzmA activity and assess its potential as a biomarker of immune response during immunotherapy.^[^ ^26^ ^]^ By correlating probe signal with immune cell infiltration and activation status, such probes may provide real‐time insights into treatment efficacy. The excellent S/N ratio of **5** supports the detection of GzmA activity at low concentrations, providing robust signal contrast without requiring complete enzymatic inhibition in vivo. Furthermore, covalent binding of the probe to GzmA minimizes signal diffusion and facilitates downstream gel‐based ABPP, as previously demonstrated with our GzmB‐targeting qABP.^[^ ^19^ ^]^

In addition to **5**, cell impermeable qABP **6**, which retains excellent reactivity and selectivity for GzmA, may be well positioned for specifically studying extracellular roles of GzmA in the context of inflammation and cancer. Although not clear, the limited cell permeability of **6** may stem from the steric or conformational effects introduced by the side chains of non‐natural amino acids. An alternative fluorophore/quencher pair may impart a more balanced hydrophilicity/hydrophobicity for enhanced cell permeability, whose improved serum stability could result in further reduction of background signals arising from nonspecific probe hydrolysis.

Together, these chemical tools represent an advancement for the study of GzmA biology. They enable selective detection of enzymatic activity in native environments and will facilitate a deeper understanding of the diverse roles GzmA plays in antitumor immunity and inflammation.

## Supporting Information

The authors have cited additional references within the Supporting Information.^[^ ^27^ ^,^ ^28^ ^]^

## Conflict of Interest

The authors declare no conflict of interest

## Supporting information

Supplementary Material

## Data Availability

The data that support the findings of this study are available in the supplementary material of this article.
