# Supplementary material for: Development of Granzyme A Turn‐ON Fluorescent Activity‐Based Probes
Source: Chembiochem. 2025 Nov 19;26(24):e202500771. doi: 10.1002/cbic.202500771 (PMC12703428; doi:10.1002/cbic.202500771)
Supplement: Supplementary file 1 — Supplementary Material [file CBIC-26-e202500771-s001.pdf]

Supplementary Information

Development of granzyme A turn-ON fluorescent activity-based probes

Sebastian M. Malespini, Muhammad Kazim, Euna Yoo*

Chemical Biology Laboratory, Center for Cancer Research, National Cancer Institute, National Institutes of Health, Frederick, Maryland 21702, United States

*Correspondence: euna.yoo@nih.gov

Supplementary Figures

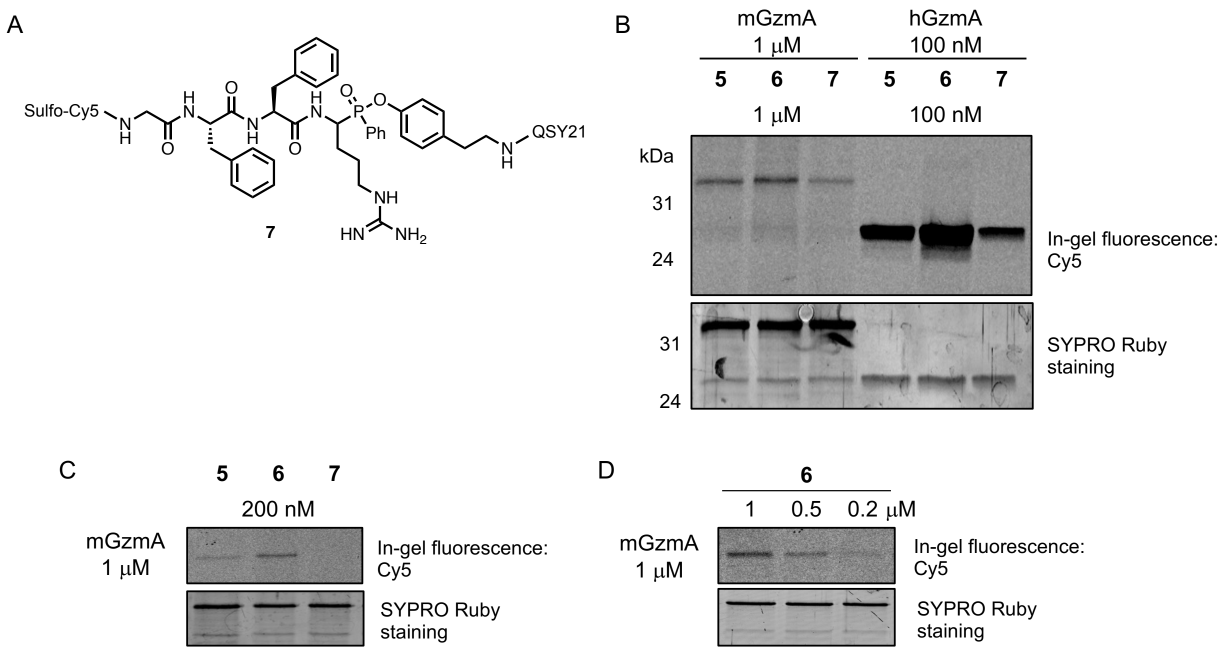

Supplementary Figure 1. (A) Chemical structure of qABP **7**. (B) Labeling of mGzmA (1 μM, 24 h, 37 °C) and hGzmA (100 nM, 2 h, 37 °C) by 1 μM or 100 nM of **5**, **6**, and **7**, respectively. (C) Labeling of mGzmA (1 μM, 24 h, 37 °C) by 200 nM of **5**, **6**, and **7**. (D) Concentration-dependent labeling of mGzmA (1 μM, 24 h, 37 °C) by **6**. Protein samples were analyzed by SDS-PAGE and in-gel fluorescence scanning for Cy5 signal. Protein loading was confirmed by SYPRO Ruby staining.

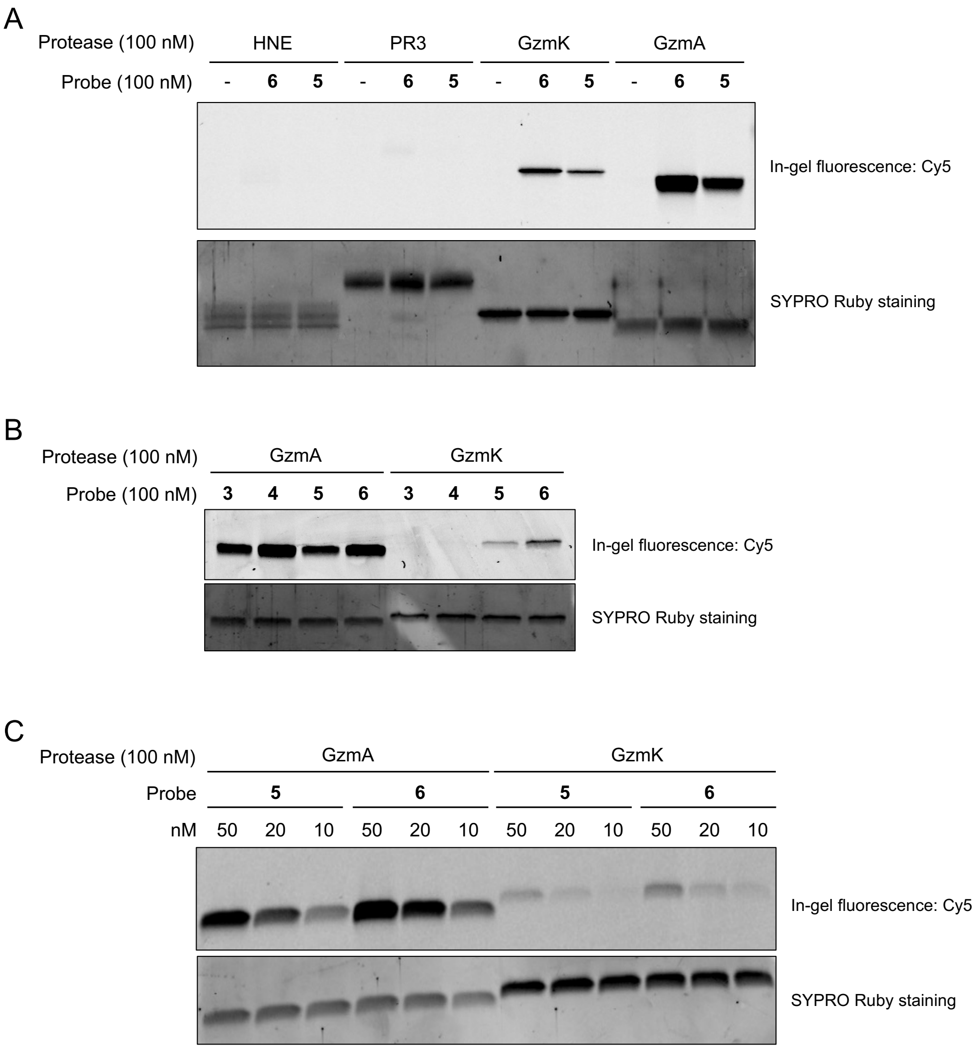

Supplementary Figure 2. Cross-reactivity of probes. (A) Labeling of GzmA, GzmK, PR3, and HNE (100 nM, 2 h, 37 °C) by 100 nM of **5** and **6**. (B) Labeling of GzmA and GzmK (100 nM, 2 h, 37 °C) by 100 nM of **3** – **6**. (C) Concentration-dependent labeling of GzmA and GzmK (100 nM, 2 h, 37 °C) by **5** and **6.** Protein samples were analyzed by SDS-PAGE and in-gel fluorescence scanning for Cy5 signal. Protein loading was confirmed by SYPRO Ruby staining.

A

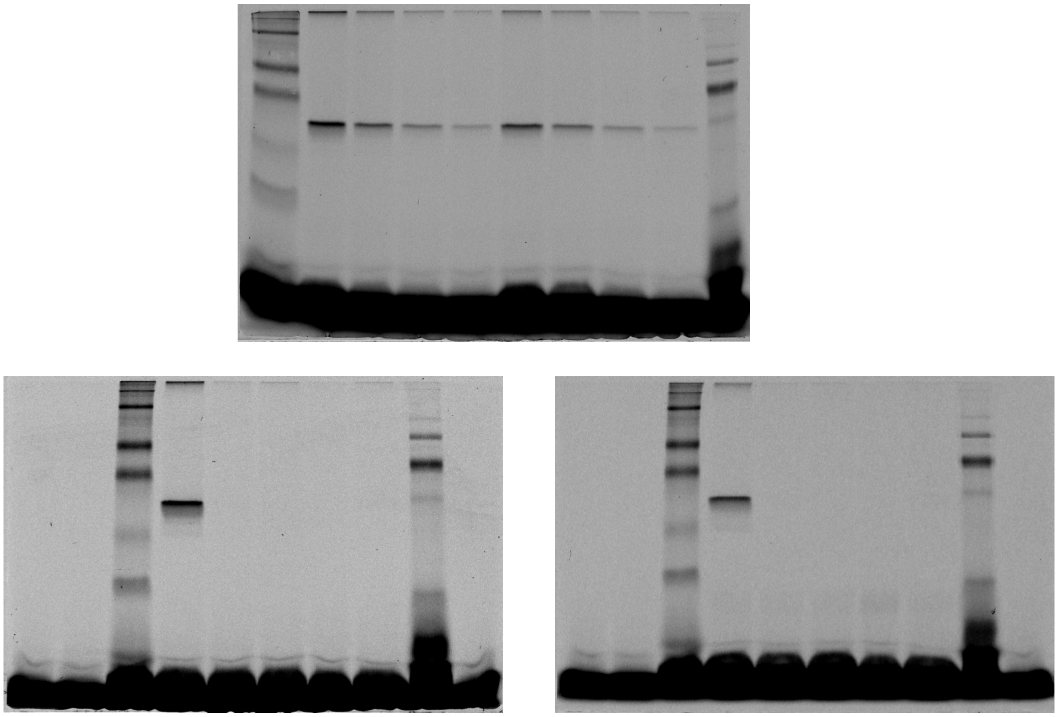

B

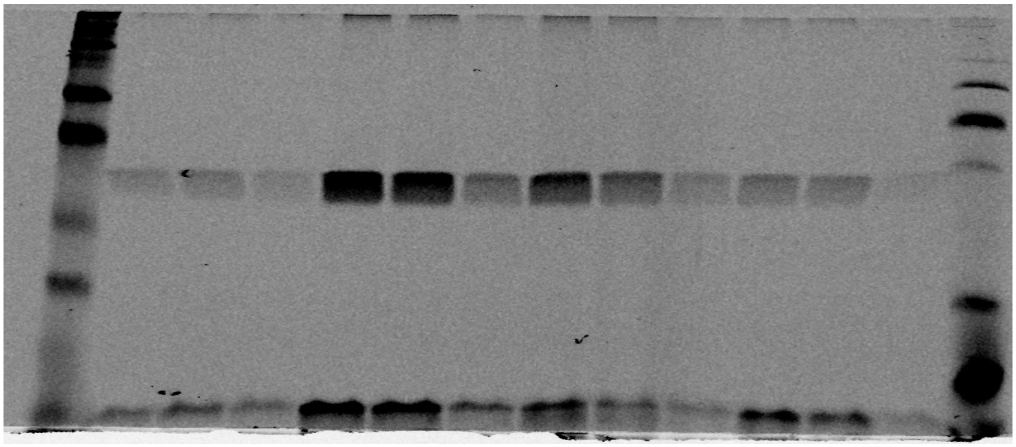

Supplementary Figure 3. Uncropped gel images for (A) Figure 4A and (B) Figure 4C.

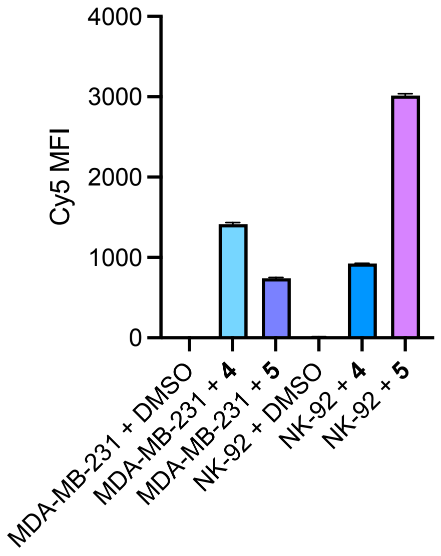

Supplementary Figure 4. S/N comparison between **4** and **5**. Flow cytometry analysis of protein labeling by ABPs **4** and **5** (5 μM, 2 h, 37 °C) in NK-92 and MDA-MB-231 cells. Data points are displayed as mean ± SD (n = 3).

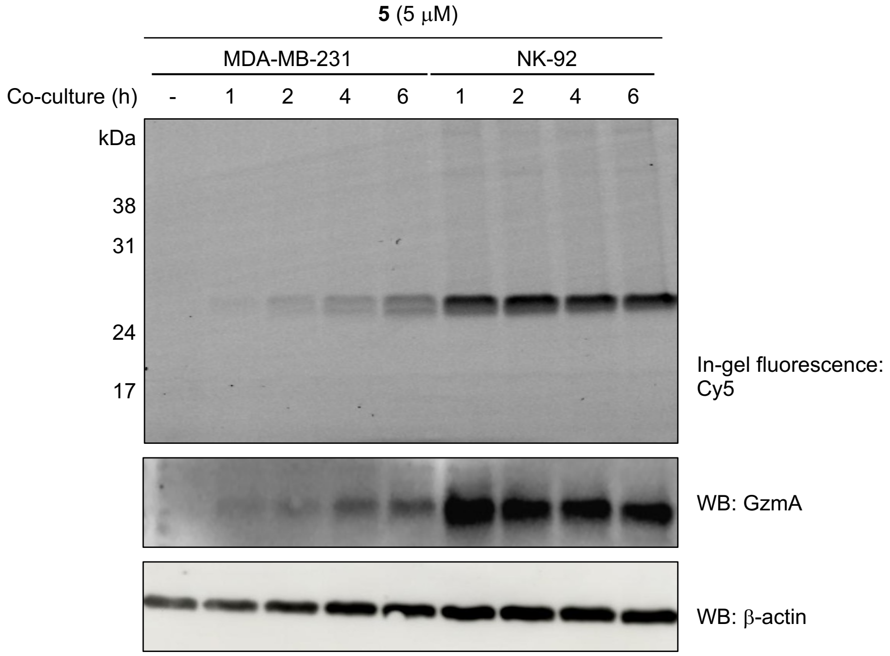

Supplementary Figure 5. Transfer of GzmA to MDA-MB-231 cells after incubation with NK-92 cells. $5x10^5$ MDA-MB-231 cells were plated and cultured overnight, then co-cultured with $2x10^6$ NK-92 cells in the presence of qABP **5** (5 μM) for the indicated time. NK-92 cells were removed, and MDA-MB-231 cells were washed, harvested, and lysed. Protein samples were analyzed by SDS-PAGE and in-gel fluorescence scanning for Cy5 signal. Expression of GzmA was confirmed by western blotting. Protein loading was confirmed with western blotting for β-actin.

Methods

A. Granzyme A cleavage assay

Briefly, hGzmA (50 nM) was incubated with either inhibitor **1** or **2** (200 nM), and GzmA fluorogenic substrate Ac-Tic-Gly-Pro-Arg-PABA-MU (100 μM) in 50 mM Tris, 150 mM NaCl, 0.01% Triton X-100 (pH 8) for 15 hr at 37 ºC. Recorded fluorescence at Ex/Em: 380/460 nm for residual GzmA proteolytic activity. Data was analyzed and processed with Prism, and data points are displayed as mean ± SD (n = 3).

B. Cell culture and preparation of cell lysates

All cell lines were cultured at 37 °C under 5% CO_2 in a humidified atmosphere. Jurkat (A3, ATCC Cat. No. CRL-2570) and 4T1 (ATCC Cat. No. CRL-2539) cells were cultured in RPMI supplemented with 10% fetal bovine serum (FBS), 1% penicillin/streptomycin (PS), and 2 mM L-glutamine, while HEK293T and MDA-MB-231 cells (ATCC Cat. No. HTB-26) were cultured in DMEM supplemented likewise as described above. NK-92 cells (ATCC Cat. No. CRL-2407) were cultured in Myelocult™ H5100 media supplemented with Gibco™ Horse Serum (63 mL/500 mL media; New Zealand Origin), penicillin/streptomycin (1% of culture media), and IL-2 (1 mL/500 mL media). To prepare the lysates, cells were harvested, pelleted (125g, 6 min), washed with ice-cold PBS, resuspended in ice-cold RIPA (40 μL/1×10^6 cells) on ice for 30 min, and pelleted by ultracentrifugation (15000g, 30 min, 4 °C). The supernatant was collected, and protein concentration was measured using a Pierce BCA protein assay. Protein concentrations were adjusted to 1 mg/mL and stored at -80 °C (or used for further analysis).

C. In-gel fluorescence imaging

For recombinant enzyme labeling, working solutions of hGzmA (purchased from R&D Systems, Cat. No. 2905-SE-010 for pro-from and Enzo Life Sciences, Cat. No.: ALX-201-118-C010 for active, mature form) were prepared by diluting a 12.6 μM stock in the following assay buffer: 50 mM Tris, 100 mM NaCl, 5 mM $CaCl_2$, 0.01% (v/v) Tween-20, pH 7.5. Working solutions of mGzmA (purchased from Cusabio Cat. No.: CSB-EP010081MO for active, mature form) were prepared by diluting a 12.6 μM solution in storage buffer in 0.1 M Tris, pH 8. In-gel fluorescence and SYPRO-Ruby staining images were acquired on a Typhoon FLA9500 instrument, while western blot images were acquired on an AMHERST ImageQuant 800 instrument.

Conditions for probe/inhibitor treatment for in-gel fluorescence analysis: Inhibitors and probes were incubated with active (or pro-) GzmA followed by treatment with 4X SDS reducing sample buffer. Samples were then heated to 100 °C for 5 min, brought to room temperature, and centrifuged. 20 μL of each sample was loaded onto a 15% polyacrylamide gel, which was then subjected to electrophoresis followed by the acquisition of in-gel fluorescence images. GzmA labeling was confirmed by western blotting, while protein loading was confirmed with SYPRO-Ruby staining. Incubation conditions for each figure/experiment are described below:

Conditions for Figure 3A: For labeling comparison between **3, 4,** and **5**, 100 nM of human GzmA was incubated with various concentrations of each probe for 2 h at 37 °C. For time-dependent labeling, 50 nM of **5** was incubated with 100 nM of human GzmA at 37 °C for the specified durations. Final reaction volume for both was 20 μL.

Conditions for Figure 3B: 100 nM of human GzmA was incubated with DMSO or 5 μM of **1** or **2** for 2 h at 37 °C in Eppendorf tubes. 0.5 μL of a 2 μM solution of **3, 4,** or **5** in DMSO was then spiked into the Eppendorf tube, yielding a final probe concentration of 50 nM. The solution was incubated for another 2 h at 37 °C. Final reaction volume was 20 μL.

Conditions for Figure 3E: 100 nM of human GzmA was incubated with various concentrations of **5** or **6**, respectively, for 2 h at 37 °C. Final reaction volume was 20 μL.

Conditions for Figure 3F: 1 μM of mouse GzmA was incubated with 1 μM of **5** or **6**, respectively, for 24 h at 37 °C. Final reaction volume was 20 μL.

Conditions for Figure 4A: 1 mg/mL of lysates was incubated with various concentrations of **5** or **6**, respectively, for 1 h at 37 °C.

Conditions for Figure 4C: Various concentrations of probes **3**-**6** were incubated with $1x10^6$ NK-92 cells for 2 h at 37 °C in a humidified incubator. Cells were centrifuged, washed with PBS (x2) and lysed with ~40 μL of ice-cold RIPA buffer. Lysates were collected, protein concentration was measured with PIERCE BCA protein assay and samples were analyzed with in-gel fluorescence and GzmA western blotting.

D. Flow cytometry analysis

All flow cytometry data were collected on BD LSRFortessa and FACSymphony A5 Cell Analyzers and analyzed with FlowJo software. Briefly, cells were processed according to the intended protocols for each experiment. Thereafter, cells were pelleted, fixed in the dark for 20 min at room temperature (Fixation buffer: BioLegend #420801), and pelleted again by centrifugation. Fixed cells were then resuspended in 1x intracellular staining perm wash buffer (BioLegend #421002), followed by centrifugation two times. Fixed/Permeabilized cells were then resuspended in appropriate amounts of cell staining buffer (BioLegend #420201) and analyzed with flow cytometry and FlowJo software.

Conditions for Figure 4D and 4E: In 12x75 mm tubes, $1x10^6$ NK-92 cells were suspended in 500 μL of fresh media and treated with either DMSO or varying concentrations of probes **3**, **4** and **5**. For S/N determination, $5x10^5$ MDA-MB-231 cells were treated with DMSO or 5 μM qABP **5** in 12x75 mm tubes. Mixtures were incubated at 37 °C for 2 h under 5% CO_2 in a humidified atmosphere. Thereafter, cells were pelleted, fixed, permeabilized, and analyzed with Flow cytometry according to the procedure outlined above.

Conditions for Figure 4F: In a 6-well cell culture plate, $5x10^5$ MDA-MB-231 cells were seeded and allowed to attach overnight. After confirming cell attachment under the microscope, media were removed and fresh media containing $2x10^6$ NK-92 cells (or NK-92 culture media alone for control) were added along with simultaneous addition of qABP **5** (5 μM). Cells were co-cultured for the indicated times at 37 °C under 5% CO_2 in a humidified atmosphere. Suspended cells (NK-92 cells) were carefully removed and collected in 12x75 mm tubes, washed with PBS (x2), fixed, permeabilized, and analyzed with flow cytometry according to the procedure outlined above. The attached cells (MDA-MB-231 cells) were carefully washed several times with PBS, detached by trypsinization, and collected in 12x75 mm tubes. Cells were washed with PBS, then fixed, permeabilized, and analyzed with flow cytometry as described above.

E. Synthesis of compounds

^1H, ^{13}C and ^{31}P spectra were acquired on a 500 MHz NMR in $CDCl_3$ or CD_3OD at 25 °C. The ^1H chemical shifts are given in parts per million (δ) with respect to an internal tetramethylsilane (TMS, δ 0.00 ppm) standard. NMR data are reported in the following format: chemical shifts (multiplicity (s = singlet, d = doublet, t = triplet, m = multiplet), coupling constants [Hz], integration). LCMS data were acquired on Agilent 1200 series LC/MS or Shimadzu Prominence-i LC-2030s instrument, whereas HRMS data were obtained on an Agilent Q-TOF LC/MS/MS instrument. All compounds are >95% pure by HPLC analysis.

Synthesis of intermediate peptides:

Intermediate peptides were synthesized through solid-phase peptide synthesis (SPPS) following a subsequent Fmoc-deprotection strategy. Briefly, 2-chlorotrityl resin was first swelled in DCM for 30 minutes in a SPPS vessel. After flushing the DCM, solution of P1 amino acid (3 eq) and collidine (5 eq) in DCM was added and the vessel was rocked overnight on a shaking platform at room temperature (Note: small amounts of DMF were added if the P1 amino acid showed solubility issues). After flushing the solution, resin was washed with DCM (x2), 6 mL of blocking agent (DCM:MeOH:DIPEA = 17:2:1) was added and the vessel was rocked at room temperature for 30 minutes. After flushing the solution, resin was washed sequentially with DCM (x2) and DMF (x2). Thereafter, subsequent Fmoc deprotection and amino acid coupling reactions were performed to build the intermediate peptide (coupling conditions: amino acid = 3 eq; HCTU = 5 eq; 2,4,6-collidine = 5 eq). After solid-phase synthesis was completed, peptide was cleaved with 20% hexafluoroisopropanol (HFIP) in DCM for 30 min (x2). The collected mixture was dried under reduced pressure, purified with reverse phase HPLC, fractions containing intermediate peptides were lyophilized and characterized with LCMS.

Synthesis of GzmA ABPs:

Compounds I-7 and I-8: The phosphonate and phosphinate cores **I-7** and **I-8** were synthesized following previously reported protocols.[1-2] Briefly, intermediate **I-1** obtained by the protection of phthalic anhydride with 4-aminobutanol was oxidized to the aldehyde **I-2** by Swern oxidation followed by a 3-component reaction with benzyl carbamate and triphenyl phosphite or dichlorophenyl phosphine to form the corresponding phosphonate (**I-3**) or phosphinate (**I-4**) intermediates. Selective deprotection of the phtalimide moiety generated the intermediate amines **I-5** and **I-6** before treatment Bis-Boc-pyrazolocarboxamidine to form **I-7** and **I-8**.

Compound I-9. Compound **I-9** was synthesized following previously reported protocols and its analytical data matched with previous reports.[2] LCMS m/z $[M+H]^+$ Calcd for $C_{17}H_{24}N_4O_3P^+$ 363.2; Found 363.2.

Compound I-10. 470 mg (0.69 mmol, 1 eq) of **I-8** was dissolved in 3.5 mL of 33% HBr in AcOH and allowed to stir at room temperature for 2 h. Excess reagents were removed under air, and the crude was suspended in cold ether. After decanting the mother liquor, the precipitates obtained were subjected to separation by reverse-phase HPLC. Purified fractions were lyophilized, yielding 102 mg (43%) of a white solid. ^{1}H NMR (500 MHz, MeOD) δ 7.99 (ddd, J = 12.4, 8.1, 1.4 Hz, 2H), 7.76 (td, J = 7.4, 1.4 Hz, 1H), 7.65 (td, J = 7.6, 3.8 Hz, 2H), 7.31 (qd, J = 7.1, 2.0 Hz, 2H), 7.24 – 7.13 (m, 3H), 4.18 (ddd, J = 10.3, 8.0, 5.6 Hz, 1H), 3.30 (d, J = 5.0 Hz, 2H), 2.21 (dtt, J = 14.8, 11.8, 5.8 Hz, 1H), 2.11 – 1.92 (m, 2H), 1.86 (dtt, J = 18.9, 13.1, 6.2 Hz, 1H).

Ac-Ile-Gly-Asn(Trt)-OH (Compound I-11): Tripeptide **I-11** was synthesized through solid-phase peptide synthesis following general procedure outlined above. LCMS m/z: $[M + H]^+$ Calcd for $C_{33}H_{39}N_4O_6^+$ 587.3; Found 587.3.

Compound 1. 35 mg (0.060 mmol, 1 eq) of **I-11**, 25 mg (0.066 mmol, 1.1 eq) of HATU, and 39.3 μL (0.30 mmol, 5 eq) of 2,4,6-trimethylpyridine were added to a 20 mL scintillation vial containing 26 mg (0.072 mmol, 1.2 eq) of **I-9** dissolved in 3 mL of DMF. The reaction was allowed to stir at room temperature for 2 h, after which solvent was removed under vacuum. The crude was taken forward to the next step, in which 3 mL of a 2.5% DCM, 2.5% TIPS, 95% TFA solution was added and stirred for an hour. Excess reagents were removed under air and mixture was purified by reverse-phase HPLC. Desired fractions were collected and lyophilized, yielding 3.63 mg (9%) of a white solid. HRMS (Q-TOF) m/z: $[M+H]^+$ Calcd for $C_{31}H_{46}N_8O_8P^+$ 689.3171; Found 689.3183.

Compound 2. 53 mg (0.09 mmol, 1 eq) of **I-11**, 37.6 mg (0.099 mmol, 1.1 eq) of HATU, and 59.5 µL (0.45 mmol, 5 eq) of 2,4,6,-trimethylpyridine were added to a 20 mL scintillation vial containing 37.5 mg (0.11 mmol, 1.2 eq) of **I-10** dissolved in 3 mL of DMF. The reaction was allowed to stir for 2 h, after which solvent was removed under vacuum. The crude was taken forward to the next step, in which 3 mL of a 2.5% DCM, 2.5% TIPS, 95% TFA solution was added and stirred for an hour. Excess reagents were removed under air and mixture was purified by reverse-phase HPLC. Desired fractions were collected and lyophilized, yielding 5.34 mg of a white solid (13%). HRMS (Q-TOF) m/z: $[M+H]^+$ Calcd for $C_{31}H_{46}N_8O_7P^+$ 673.3222; Found 673.3231.

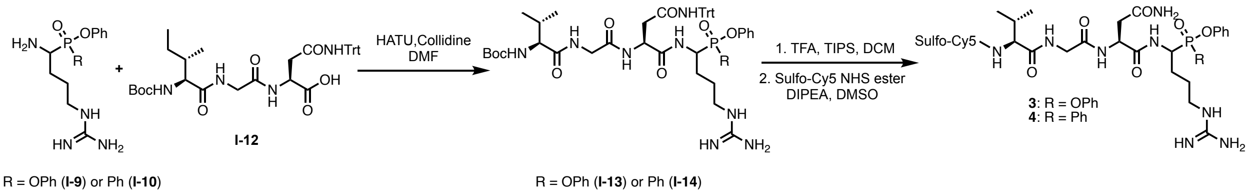

Scheme S1. Synthesis of phosphonate and phosphinate GzmA ABPs **3** and **4**.

Boc-Ile-Gly-Asn(Trt)-OH (Compound I-12): Tripeptide **I-12** was synthesized through solid-phase peptide synthesis following general procedure outlined above. LCMS m/z: $[M + H]^+$ Calcd for $C_{36}H_{45}N_4O_7^+$ 645.3 Found 645.3.

Compound I-13. 31.8 mg of **I-9** (0.084 mmol, 1.1 eq) was added to a solution of 48.9 mg of **I-12** (0.076 mmol, 1 eq) in 2 mL of anhydrous DMF. After addition of 32 mg HATU (0.084 mmol, 1.1 eq) and 50.8 µL of 2,4,6-trimethylpyridine (0.39 mmol, 5 eq), the reaction was allowed to run for 4 h, and progress was monitored by LC-MS. Solvent was removed, and the crude was purified by reverse-phase HPLC. Desired fractions were collected and lyophilized, yielding 58 mg of a white solid (77%). LCMS m/z $[M+H]^+$ Calcd for $C_{53}H_{66}N_8O_9P^+$ 989.5; Found 990.3.

Compound I-14. 32 mg of **I-10** (0.0935 mmol, 1.1 eq) was added to a solution of 54.5 mg of **I-12** (0.085 mmol, 1 eq) and 35.5 mg of HATU (0.0935 mmol, 1.1 eq) in 2 mL of anhydrous DMF. After the addition of 56.2 µL of 2,4,6-trimethylpyridine (0.43 mmol, 5 eq.), the reaction was allowed to run for 4 h, and progress was monitored by LC-MS. Solvent was removed, and the crude was purified by reverse-phase HPLC. Desired fractions were collected and lyophilized, yielding 33 mg of a white solid (40%). LCMS m/z $[M+H]^+$ Calcd for $C_{53}H_{66}N_8O_8P^+$ 973.4; Found 973.4.

ABP 3. 22.5 mg of **I-30** (0.023 mmol, 1 eq) was dissolved in a solution of 10:1:1 DCM: TFA: TIPS and allowed to stir for 5 h with reaction progress monitored by LCMS. Excess reagents were removed under vacuum, and the crude was taken forward to the next step without purification. After dissolving the crude in 1 mL of anhydrous DMSO, 4.3 mg (0.0057 mmol, 0.25 eq) of Sulfo-Cy5-NHS ester and 40 µL (0.23 mmol, 10 eq) of DIPEA were added. The reaction vessel was covered in aluminum foil and allowed to stir overnight at room temperature under N_2. Purification was carried out by reverse-phase HPLC, yielding 1.85 mg (25%) of a blue solid. HRMS (Q-TOF) m/z: $[M+2H]^{2+}$ Calcd for $C_{62}H_{83}N_{10}O_{14}PS_2^{2+}$ 643.2629; Found 643.2651 (Deconvoluted 1284.51).

ABP 4. 16.5 mg of **I-31** (0.017 mmol, 1 eq) was dissolved in a solution of 10:1:1 DCM: TFA: TIPS and allowed to stir for 3 h with reaction progress monitored by LCMS. Excess reagents were removed under vacuum, the crude was dissolved in 1 mL of anhydrous DMSO, and 9 mg (0.012 mmol, 0.70 eq) of Sulfo-Cy5-NHS ester and 30 µL (0.17 mmol, 10 eq) of DIPEA were added. The reaction vessel was covered in aluminum foil and allowed to stir overnight at room temperature under N_2. Purification was carried out by reverse-phase HPLC, yielding 1.16 mg (7.6%) of a blue solid. HRMS (Q-TOF) m/z: $[M+2H]^{2+}$ Calcd for $C_{62}H_{83}N_{10}O_{13}PS_2^+$ 635.2655; $Found^+$ 635.2674 (Deconvoluted 1268.52).

Compound I-15. 3.34 g (6.8 mmol, 1 eq) of the intermediate phosphinic acid was dissolved in 15 mL of toluene. 1.65 g (7.48 mmol, 1.1 eq) of alloc-tyramine, 4.23 mL DIC (27.2 mmol, 4 eq), and 83.1 mg (0.68 mmol, 0.1 eq) of DMAP were added, a condenser was attached, and the reaction was run overnight at 80 °C. Solvent was then removed, and the crude was suspended in DCM and subsequently washed x3 with each of the following: 1M $KHSO_4$, H_2O, $NaHCO_3$, and brine. Organic layer was dried over anhydrous Na_2SO_4, evaporated under reduced pressure, and subjected to purification with combi-flash silica gel chromatography, yielding 2.61 grams (45%) of yellow oil. 1H NMR (400 MHz, MeOD) δ 7.89 – 7.79 (m, 6H), 7.63 – 7.55 (m, 1H), 7.47 (tt, J = 8.0, 3.9 Hz, 2H), 7.34 – 7.21 (m, 4H), 7.19 – 7.14 (m, 1H), 7.11 – 6.95 (m, 4H), 5.90 (ddt, J = 16.2, 10.7, 5.4 Hz, 1H), 5.29 – 5.23 (m, 1H), 5.16 (dd, J = 10.5, 1.6 Hz, 1H), 5.07 – 4.91 (m, 2H), 4.66 – 4.54 (m, 1H), 4.49 (d, J = 5.4 Hz, 2H), 3.88 – 3.65 (m, 2H), 3.25 (t, J = 7.3 Hz, 2H), 2.70 (t, J = 7.3 Hz, 2H), 2.01 – 1.84 (m, 2H), 1.79 (q, J = 8.6 Hz, 2H), 1.14 (dd, J = 20.8, 6.3 Hz, 2H). LCMS m/z $[M+H]^+$ Calcd for $C_{38}H_{39}N_3O_8P^+$ 696.2; Found 696.5.

Compound I-16. 1.2 g (1.73 mmol, 1 eq) of **I-15** was dissolved in 17 mL of EtOH, 347 μL (5.19 mmol, 3 eq) of ethylenediamine was added, and the reaction was heated for 78 °C for 2 h. Upon complete consumption of the starting material, monitored by LC-MS and TLC, the reaction was allowed to cool to room temperature, and solvent was removed under reduced pressure. After filtering precipitates, the filtrate was dried under reduced pressure, yielding a yellow oil that was taken to the next step without purification. The presence of product was confirmed by LCMS m/z $[M+H]^+$ Calcd for $C_{30}H_{37}N_3O_6P^+$ 566.2; Found 566.4.

Compound I-17. Roughly 980 mg (1.73 mmol, 1 eq) of **I-16** was dissolved in 25 mL DCM. 2.15 grams (6.92 mmol, 4 eq) of *N,N'*-Di-Boc-1H-pyrazole-1-carboxamidine and 965 μL (6.92 mmol, 4 eq) of TEA were added to the solution and the reaction was allowed to stir overnight at room temperature. Upon consumption of the starting material (LC-MS), solvent was removed under reduced pressure, and the mixture was resuspended in EtOAc. Organic layer was subsequently washed x3 with each of the following: 1N HCl, $NaHCO_3$, and brine. It was then dried over anhydrous Na_2SO_4, filtered, evaporated under reduced pressure, and subjected to purification with combi-flash silica gel chromatography, yielding 585 milligrams (42%) of yellow oil. 1H NMR (500 MHz, MeOD) δ 7.85 (ddt, J = 11.7, 8.3, 1.9 Hz, 2H), 7.61 (tt, J = 7.4, 1.4 Hz, 1H), 7.48 (tt, J = 6.4, 3.1 Hz, 2H), 7.32 – 7.25 (m, 3H), 7.19 – 6.98 (m, 6H), 5.89 (ddt, J = 16.2, 10.7, 5.4 Hz, 1H), 5.25 (dq, J = 17.3, 1.7 Hz, 1H), 5.15 (dd, J = 10.5, 1.6 Hz, 1H), 5.04 – 4.90 (m, 2H), 4.50 – 4.41 (m, 3H), 3.46 – 3.34 (m, 2H), 3.24 (t, J = 7.3 Hz, 2H), 2.70 (td, J = 7.2, 2.6 Hz, 2H), 2.12 – 2.03 (m, 1H), 1.85 – 1.73 (m, 2H), 1.70 – 1.59 (m, 1H), 1.52 (d, J = 3.2 Hz, 9H), 1.46 (d, J = 3.0 Hz, 9H). LCMS m/z $[M+H]^+$ Calcd for $C_{41}H_{55}N_5O_{10}P^+$ 808.4; Found 809.3.

Compound I-18. 250 mg (0.31 mmol) of **I-17** was cooled to 0 °C and then dissolved in approximately 2 mL of 33% HBr in AcOH. The reaction was allowed to run for 30 min, after which the solvent was removed under air while the vial was kept at 0 °C. After diluting the crude with 3 mL of 1:1 ACN:H_2O, it was subject to separation by reverse-phase HPLC. Desired fractions were lyophilized, yielding 127 mg (86%) of a white solid. ^1H NMR (500 MHz, MeOD) δ 7.95 (ddd, *J* = 12.3, 8.2, 1.4 Hz, 2H), 7.79 – 7.71 (m, 1H), 7.63 (td, *J* = 7.7, 4.4 Hz, 2H), 7.19 – 7.08 (m, 4H), 5.88 (ddt, *J* = 16.2, 10.6, 5.3 Hz, 1H), 5.24 (dt, *J* = 17.1, 1.7 Hz, 1H), 5.15 (dt, *J* = 10.5, 1.5 Hz, 1H), 4.46 (dt, *J* = 5.4, 1.6 Hz, 2H), 4.18 – 4.02 (m, 1H), 3.27 – 3.24 (m, 4H), 2.71 (td, *J* = 7.2, 3.4 Hz, 2H), 2.16 (dtd, *J* = 14.7, 11.1, 5.6 Hz, 1H), 2.01 – 1.88 (m, 2H), 1.87 – 1.78 (m, 1H). LCMS m/z $[M+H]^+$ Calcd for $C_{23}H_{32}N_5O_4P^+$ 474.2; Found 474.2.

Compound I-19. 127 mg of **I-18** (0.27 mmol, 1.1 eq) was dissolved in 4 mL DMF and 158 mg (0.245 mmol, 1 eq) of **I-12,** 103 mg (0.27 mmol, 1.1 eq) of HATU, and 163 µL (1.23 mmol, 5 eq) of 2,4,6-trimethylpyridine were added to the solution and stirred for 2.5 h. Solvent was removed under N_2, and the mixture was purified by reverse-phase HPLC, yielding 166 mg (61%) of a white solid. LCMS m/z $[M+H]^+$ Calcd for $C_{59}H_{75}N_9O_{10}P^+$ 1100.5; Found 1100.5.

Compound I-20. 20 mg (0.018 mmol, 1.5 eq) of **I-19** was dissolved in a solution of 90:5:5 TFA:DCM:TIPS and stirred at room temperature. After complete deprotection was observed, the crude was pipetted into a 15 mL Falcon tube. Precipitation was carried out with 12 mL of cold ether, the pellets were dissolved in 500 μL anhydrous DMSO and transferred to a 1.5 mL Eppendorf tube containing 9.3 mg (0.012 mmol, 1 eq) of sulfo-Cy5-NHS. Then, roughly 21 μL (0.12 mmol, 10 eq) of DIPEA was added, the reaction vessel was covered in Al-foil, and shaken overnight (Note: additional 1 eq of TSTU and 10 eq of DIPEA were added for 24 h to reactivate any hydrolyzed sulfo-Cy5 in situ). The reaction was stopped after approximately 40 h and purified by reverse-phase HPLC. Desired fractions were collected and lyophilized, yielding 6.82 mg (41%) of a dark blue solid. LCMS m/z $[M+H]^+$ Calcd for $C_{68}H_{92}N_{11}O_{15}PS_2^+$ 698.8; Found 699.1.

qABP 5. 1.6 mg (0.0014 mmol, 0.5 eq) of $Pd(PPh_3)_4$ was quickly added to a 4 mL scintillation vial containing 4 mg (0.028 mmol, 1 eq) of **I-20** in dissolved in 1 mL anhydrous DMF. 10 μL of pyrrolidine was added, and the reaction was stirred under N_2 atmosphere. Upon complete consumption of the starting material, the crude was purified by reverse-phase HPLC, the desired fractions were lyophilized, redissolved in 1 mL of DMSO, and added to a 1.5 mL Eppendorf tube containing 1.48 mg (0.0019 mmol, 0.7 eq) of QSY-21 NHS ester. Then, 4.4 μL (0.025 mmol, 9 eq) of DIPEA was added, and the reaction was allowed to stir for 1 h, after which it was subjected to reverse-phase HPLC. Lyophilization of desired fractions yielded 1.65 mg (44%) of a dark blue solid. HRMS (Q-TOF) m/z: $[M+2H]^{2+}$ Calcd for $C_{105}H_{121}N_{14}O_{17}PS_3^{2+}$ 988.8978; Found 988.8993 (Deconvoluted 1975.78).

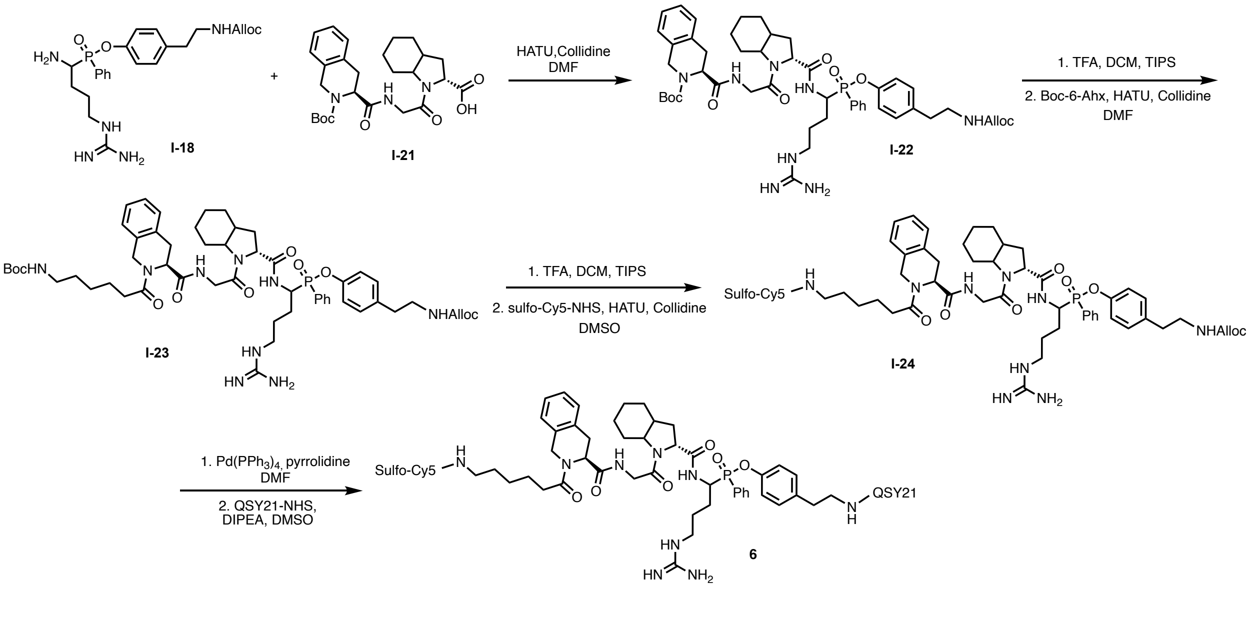

Scheme S2. Synthesis of phosphinate GzmA qABP **6**

Boc-Tic-Gly-Oic-COOH (Compound I-21): Tripeptide **I-21** was synthesized through solid-phase peptide synthesis following general procedure outlined above. LCMS m/z: $[M + H]^+$ Calcd for $C_{26}H_{36}N_3O_6^+$ 486.3; Found 486.0.

Compound I-22. 43.7 mg (0.092 mmol, 1.2 eq) of **I-18** in 2 mL of DMF was added to a 20 mL scintillation vial containing 37.4 mg (0.077 mmol, 1 eq) of **I-21** and 35 mg of HATU (0.092 mmol, 1.2 eq). Then 50.9 μL (0.385 mmol, 5 eq) of 2,4,6-trimethylpyridine was added, and mixture was stirred for 4 h. Solvent was removed under air and the crude was subjected to purification by

reverse-phase HPLC. Desired fractions were collected and lyophilized, yielding 42.2 mg (58%) of a white solid. LCMS m/z $[M+H]^+$ Calcd for $C_{49}H_{66}N_8O_9P^+$ 941.5; Found 941.2.

Compound I-23. 15.4 mg (0.016 mmol, 1 eq) of **I-22** was dissolved in 1 mL of an 18:1:1 TFA:DCM:TIPS solution and stirred at room temperature for 2 h. Excess reagents were removed under reduced pressure, and the mixture was resuspended in 1 mL of anhydrous DMF. 3.70 mg (0.016 mmol, 1 eq) of Boc-6-aminohexanoic acid, 6.1 mg (0.016 mmol, 1 eq) of HATU, and 10.6 μL (0.08 mmol, 5 eq) of 2,4,6-trimethylpyridine were added, and the reaction was stirred at room temperature. At the 1 h mark, an additional 3 mg (0.008 mmol, 0.5 eq) HATU, 1.85 mg boc-6-aminohexanoic acid (0.008 mmol, 0.5 eq) and 5.3 μL (0.04 mmol, 5 eq) of 2,4,6-trimethylpyridine were added and the mixture was stirred for another hour. The crude was concentrated under air, and diluted in 1 mL of 1:1 ACN:H_2O solution before being purified by reverse-phase HPLC. Desired fractions were collected and lyophilized, yielding 10.6 (63%) mg of a white solid. LCMS m/z $[M+H]^+$ Calcd for $C_{55}H_{77}N_9O_{10}P^+$ 1054.6; Found 1054.8.

Compound I-24. 10.6 mg (0.01 mmol, 1 eq) of **I-23** was dissolved in 1.6 mL of a 9:1:1 solution of TFA:DCM:TIPS and allowed to stir for 2.5 hours. After removing solvents under vacuum, the crude was resuspended in 2 mL of a 1:1 H_2O:ACN solution and lyophilized. It was subsequently resuspended in 1 mL of anhydrous DMSO and transferred to an Eppendorf tube containing 7.1 mg (0.01 mmol, 1 eq) of Sulfo-Cy5 NHS ester. Then, 0.065 mmol (6.5 eq) of base was added, and the reaction vessel was covered with Al-foil and allowed to stir at room temperature overnight. (Note: coupling agent and base were added for the reaction to reactivate any hydrolyzed sulfo-Cy5 in situ). The crude was purified by reverse-phase HPLC, and desired fractions were lyophilized, yielding 2.74 mg (17%) of a blue solid. LCMS m/z $[M+H]^+$ Calcd for $C_{83}H_{108}N_{11}O_{15}PS_2^+$ 796.9; Found 797.4.

qABP 6. 2.74 mg (0.0017 mmol, 1 eq) of **I-24** was dissolved in 0.5 mL of anhydrous DMF and transferred to a 4 mL scintillation vial containing 0.98 mg (0.00085 mmol, 0.5 eq) of $Pd(PPh_3)_4$. Then, 5 μL of pyrrolidine was quickly added, and the vial was sealed and put under N_2 atmosphere. After 1 hour, the reaction was stopped, and the solvent was removed under reduced pressure. The crude was purified by reverse-phase HPLC, yielding 1.82 mg (71% yield, 0.0012 mmol, 1 eq) of a blue solid that was subsequently dissolved in 500 μL of anhydrous DMSO and transferred to an Eppendorf tube containing 0.94 mg (0.0012 mmol, 1 eq) of QSY21 NHS-ester. After adding 1.05 μL (0.006 mmol, 5 eq) of DIPEA, the reaction was stirred for 1 h at room temperature. An additional 0.5 eq of QSY21 NHS-ester and 2 eq of DIPEA were added after 1 h to push conversion. After stirring for an additional hour, the crude was diluted in 500 μL of 1:1 ACN:H_2O and purified by reverse-phase HPLC. Desired fractions were lyophilized, yielding 1.13 mg (43%) of a blue solid. HRMS (Q-TOF) m/z: $[M+2H]^{2+}$ Calcd for $C_{120}H_{138}N_{14}O_{17}PS_3^{2+}$ 1086.9604; Found 1086.9632 (Deconvoluted 2171.91).

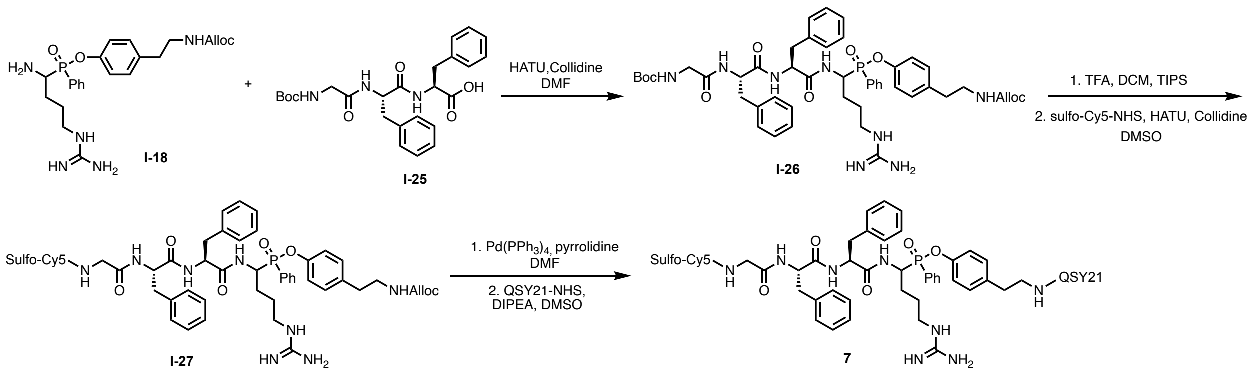

Scheme S3. Synthesis of phosphinate GzmA qABP **7**

Boc-Gly-Phe-Phe-COOH (Compound I-25): Tripeptide **I-25** was synthesized through solid-phase peptide synthesis following general procedure outlined above. LCMS m/z: $[M + H]^+$ Calcd for $C_{25}H_{32}N_3O_6^+$ 470.2, Found 470.4.

Compound I-26. 39.4 mg (0.084 mmol, 1 eq) of **I-25** was dissolved in 2 mL of DMF. 39.6 mg (0.084 mmol, 1 eq) of **I-18**, 31.9 mg (0.084 mmol, 1 eq) of HATU, and 55.5 uL (0.42 mmol, 5 eq) of 2,4,6-trimethylpyridine were added, and the reaction was allowed to run overnight at room temperature. The solvent was removed under reduced pressure, and the crude was purified by reverse-phase HPLC, yielding 49.4 mg (64%) of a white solid. LCMS m/z: $[M + H]^+$ Calcd for $C_{48}H_{62}N_8O_9^+$ 925.4, Found 926.3.

Compound I-27. 49.4 mg (0.053 mmol, 1 eq) of **I-26** was dissolved in 2 mL of a 9:1:1 TFA:DCM:TIPS solution and stirred at room temperature for 30 min. Excess reagents were removed under reduced pressure, and the crude was purified by reverse-phase HPLC. A 4.03 mg (4.9 μmol, 1 eq) aliquot of the deprotected species was subsequently dissolved in 375 uL of a DMSO solution of Cy5-OH (3.2 mg, 4.9 μmol, 1 eq) that had been pre-activated with HATU (1.86 mg, 4.9 μmol, 1 eq) and 2,4,6-trimethylpyridine (1.95 μL, 0.0147 mmol, 3 eq). The reaction was allowed to run overnight (Note: An additional 4 eq of 2,4,6-trimethylpyridine was added). Upon consumption of activated Cy5, the reaction was stopped, and the crude was purified by reverse-phase HPLC, yielding 2.4 mg of a blue solid (33%). LCMS m/z: $[M + H]^+$ Calcd for $C_{76}H_{92}N_{10}O_{14}PS_2^+$ 1463.6, Found 1464.0.

qABP 7. 2.4 mg (1.7 μmol, 1 eq) of **I-27** was dissolved in 1 mL of DMF. 0.97 mg (0.84 μmol, 0.5 eq) of $Pd(PPh_3)_4$ and 1 μL of pyrrolidine were added, and the reaction was allowed to stir under N_2 atmosphere. After 15 minutes, an additional 0.5 eq of $Pd(PPh_3)_4$ and 1 μL of pyrrolidine were added. At the 45-minute mark, the reaction was stopped, the solvent was removed under vacuum, and the crude was purified. The deprotected species was subsequently dissolved in 0.5 μL of anhydrous DMSO and added to an Eppendorf tube containing 1.3 mg (0.017 mmol, 1 eq) of QSY21 NHS ester and 0.88 μL (5.1 μmol, 3 eq) of DIPEA. The vessel was covered in aluminum foil and allowed to shake for 15 minutes, after which the crude was purified by reverse-phase HPLC, ultimately yielding 1.05 mg of a blue solid (30%). HRMS (Q-TOF) m/z: $[M+2H]^{2+}$ Calcd for $C_{113}H_{112}N_{13}O_{16}PS_3^{2+}$ 1022.4028; Found 1022.4078 (Deconvoluted 2042.80).

NMR Spectra

^{1}H NMR of Compound **I-1**

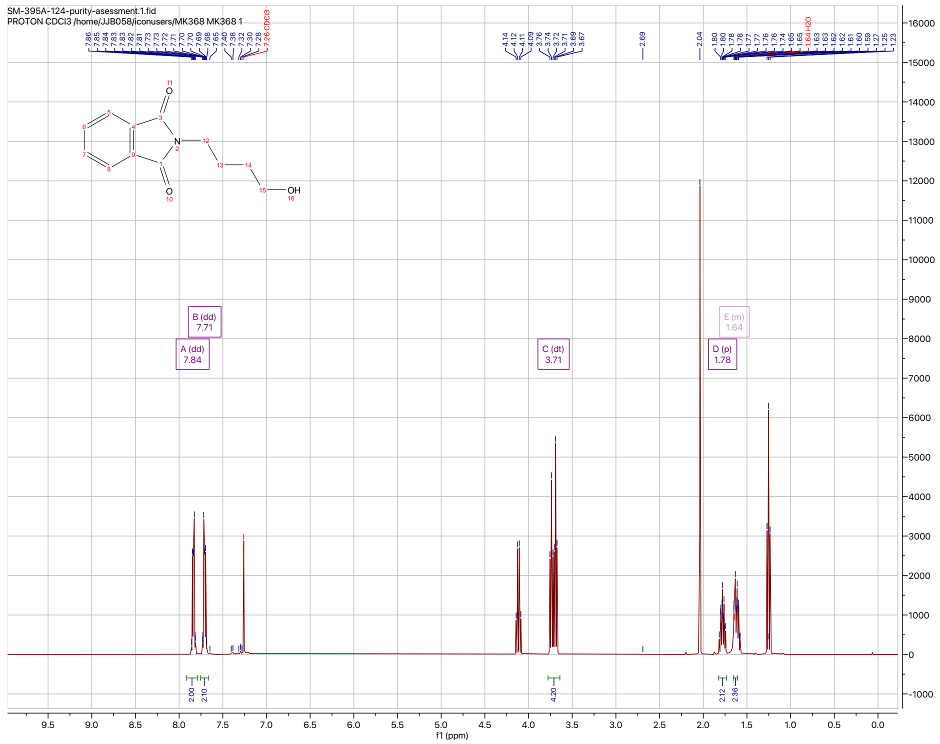

^{1}H NMR of Compound **I-2**

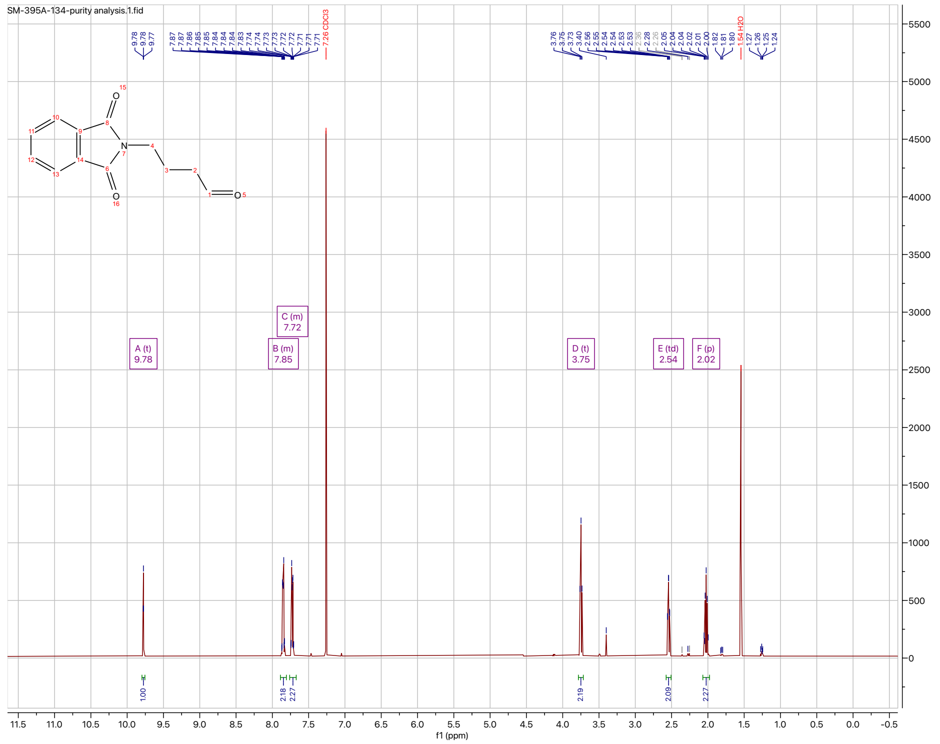

^{1}H NMR of Compound **I-3**

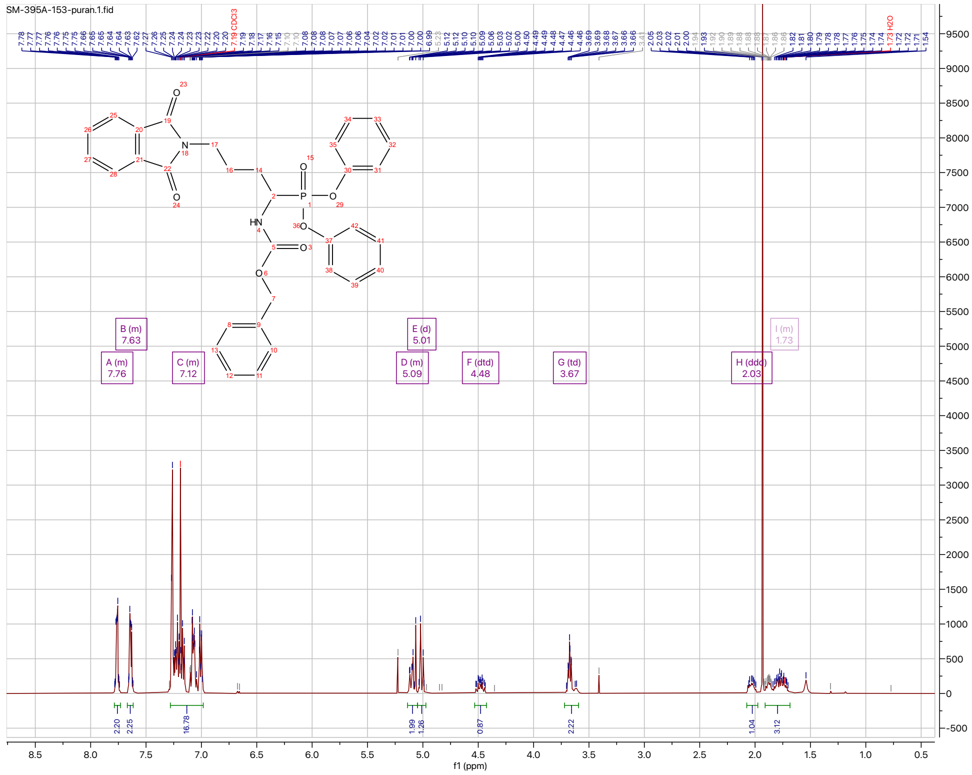

^{1}H NMR of Compound **I-9**

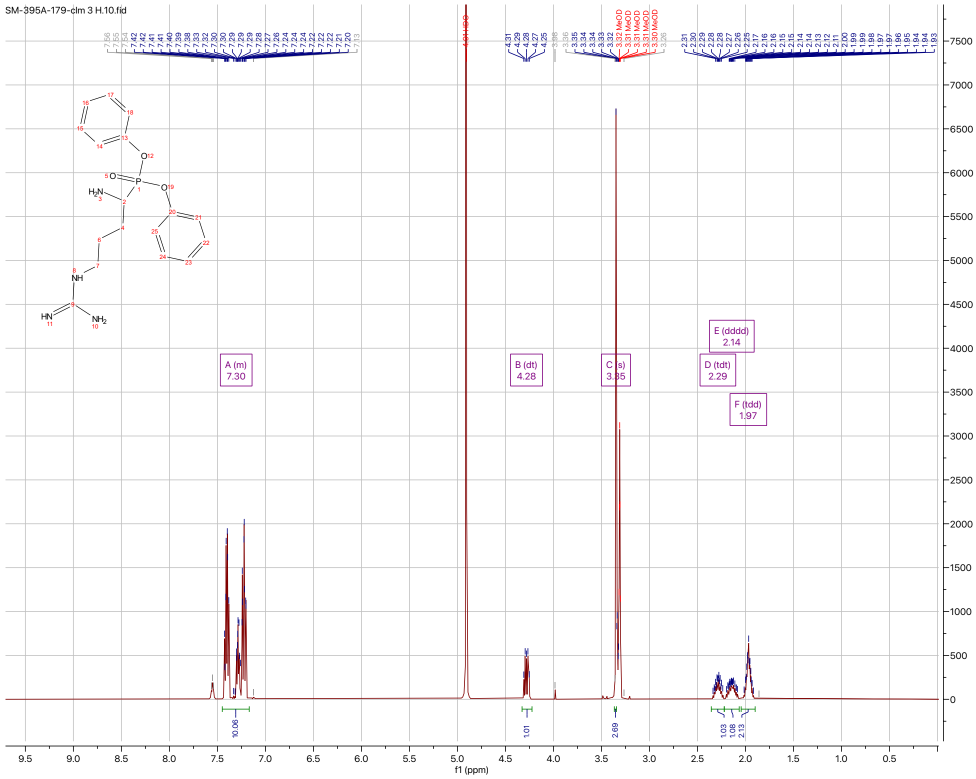

^{1}H NMR of Compound **I-10**

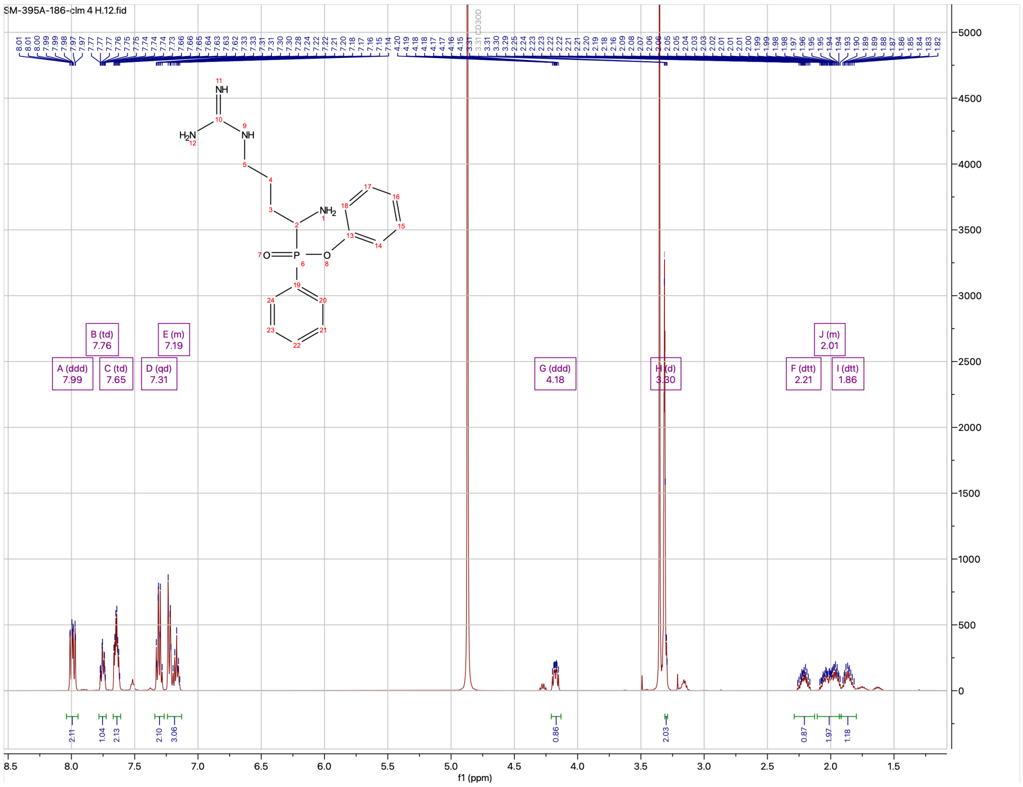

^{1}H NMR of Compound **I-15**

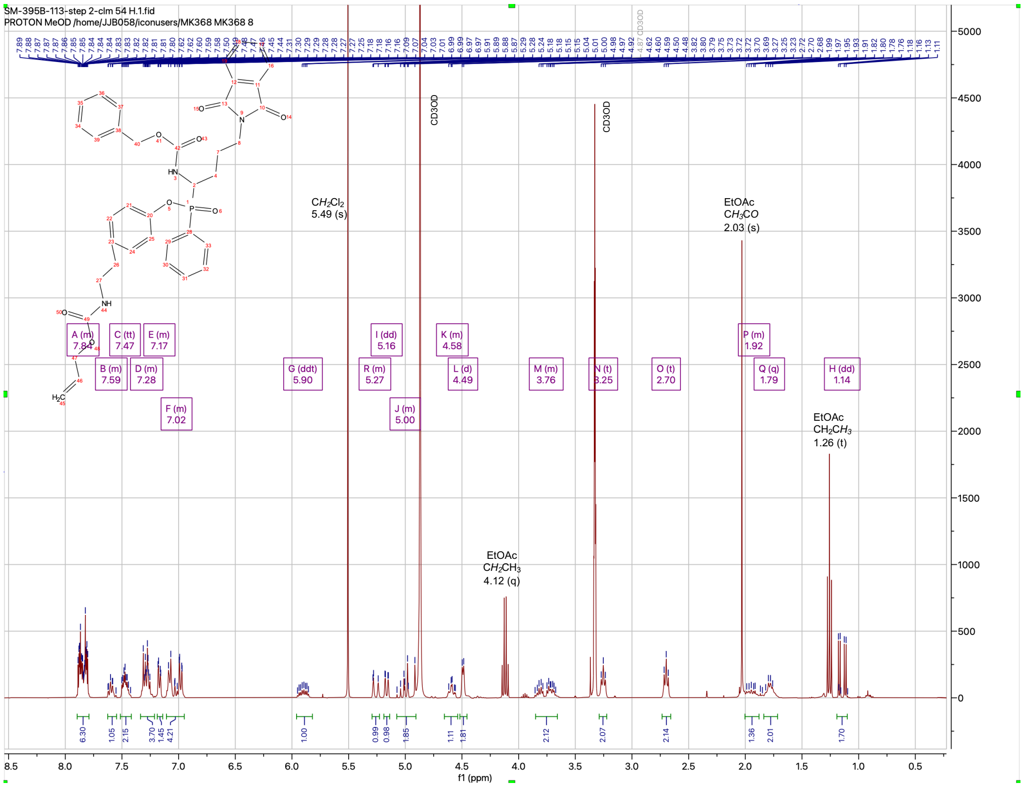

^{1}H NMR of Compound **I-17**

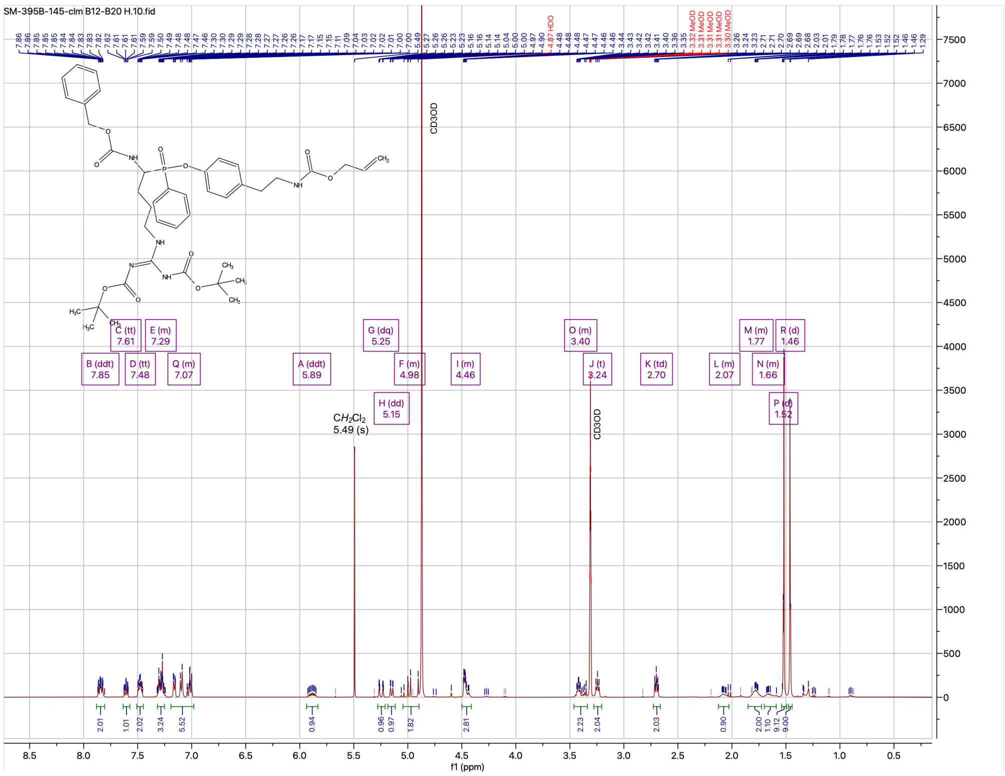

^{1}H NMR of Compound **I-18**

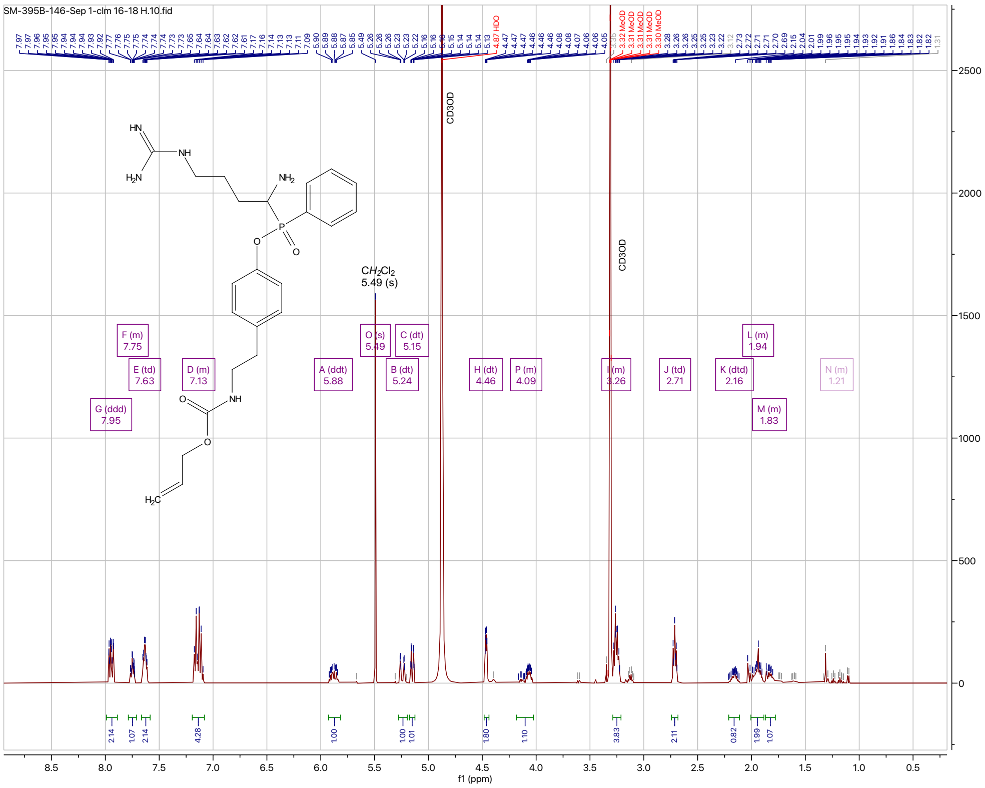

HRMS of Compound **1**

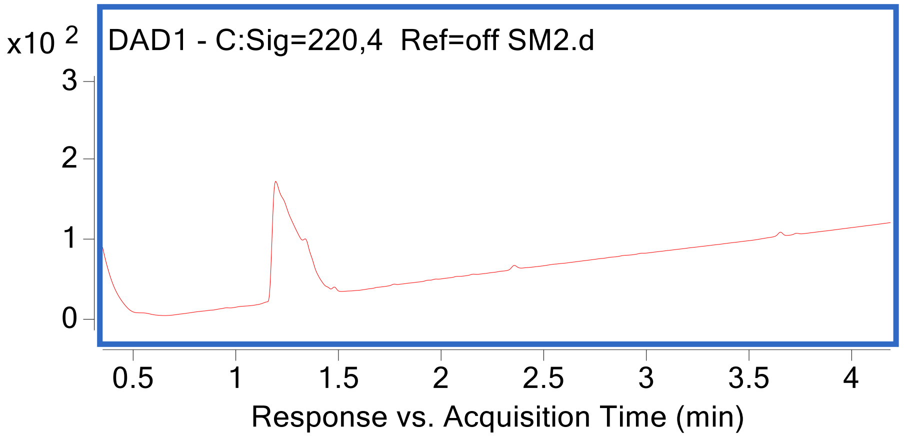

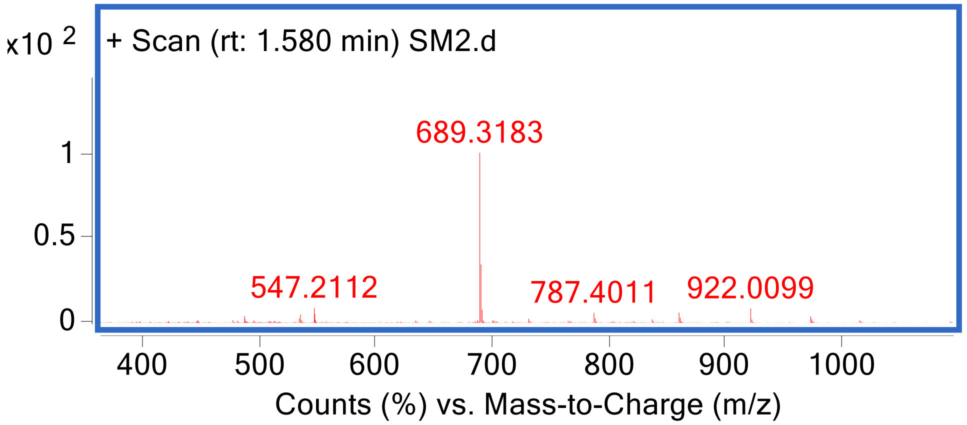

HRMS of Compound **2**

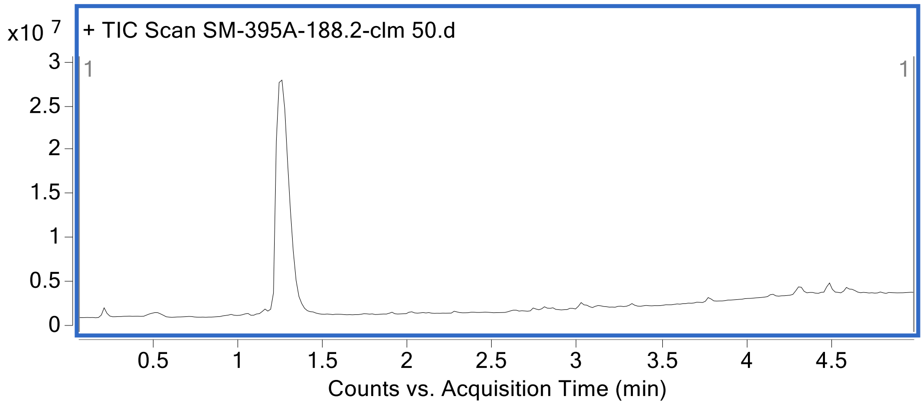

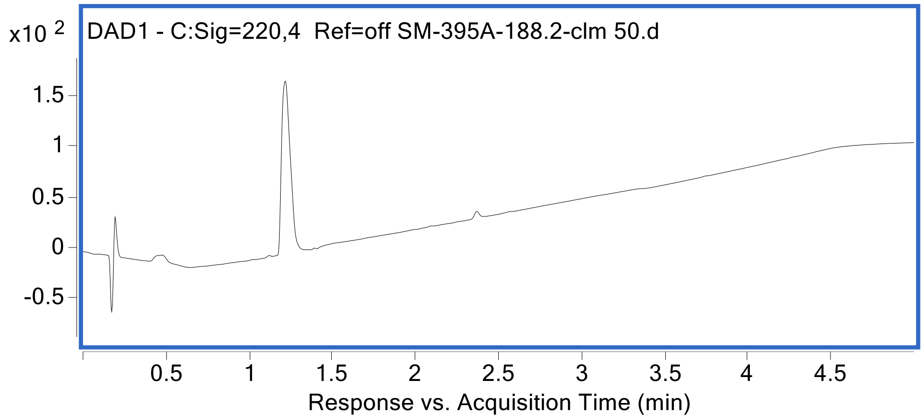

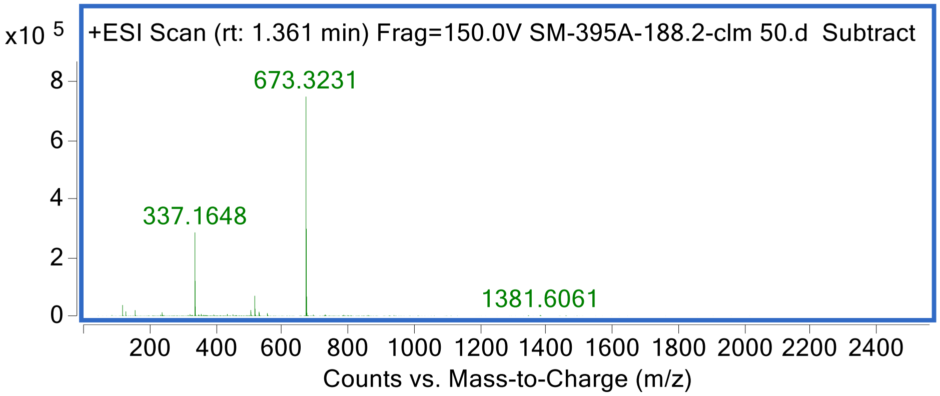

HRMS of **ABP 3**

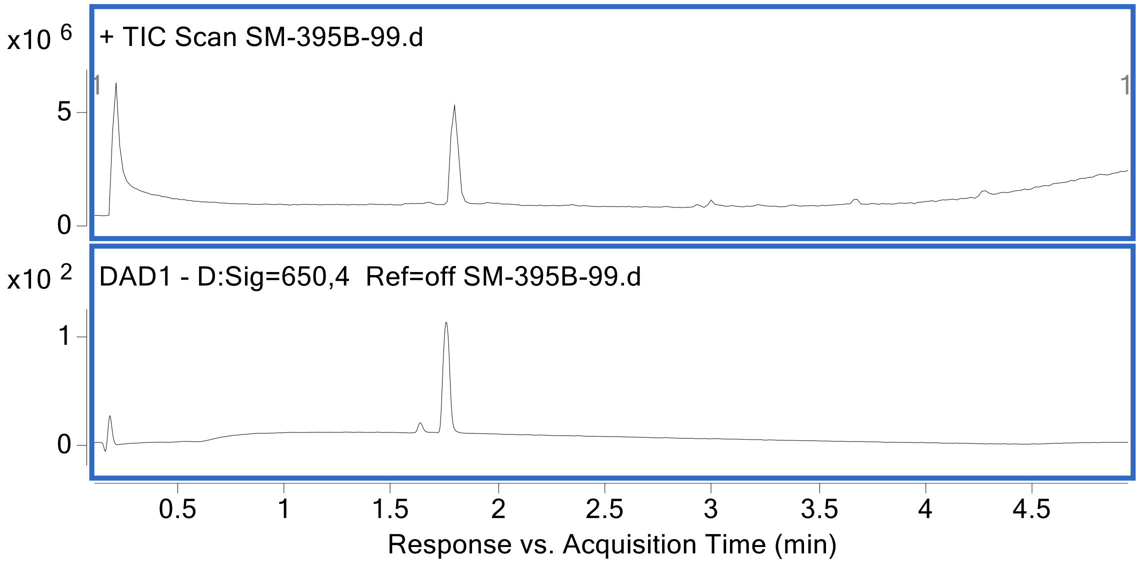
+ TIC Scan SM-395B-99.d
DAD1 - D:Sig=650,4 Ref=off SM-395B-99.d
Response vs. Acquisition Time (min)

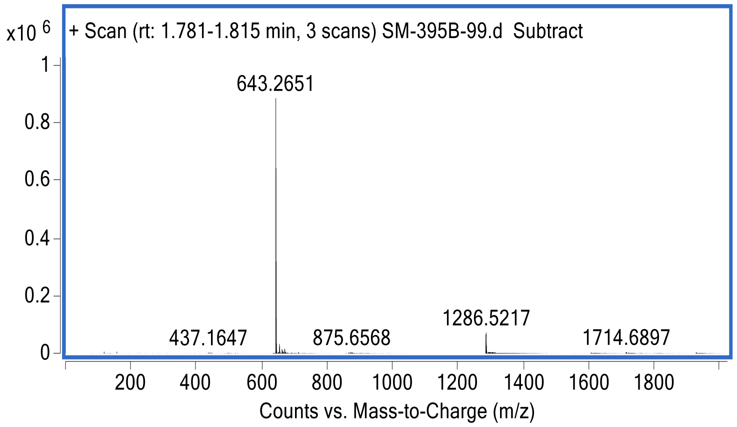
+ Scan (rt: 1.781-1.815 min, 3 scans) SM-395B-99.d Subtract
643.2651
437.1647
875.6568
1286.5217
1714.6897
Counts vs. Mass-to-Charge (m/z)

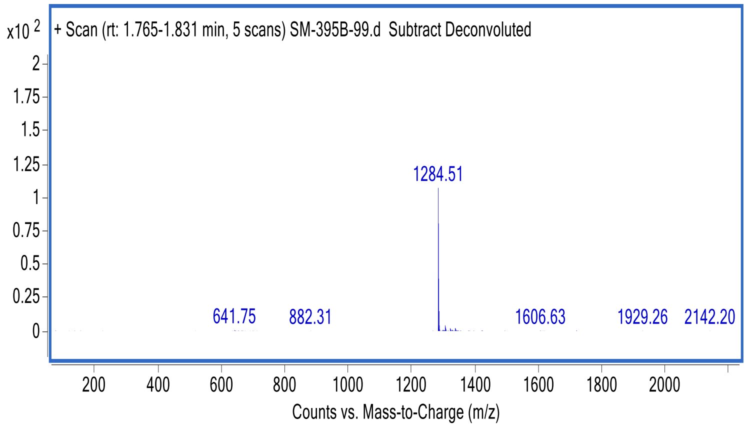
+ Scan (rt: 1.765-1.831 min, 5 scans) SM-395B-99.d Subtract Deconvoluted
1284.51
641.75
882.31
1606.63
1929.26
2142.20
Counts vs. Mass-to-Charge (m/z)

HRMS of **ABP 4**

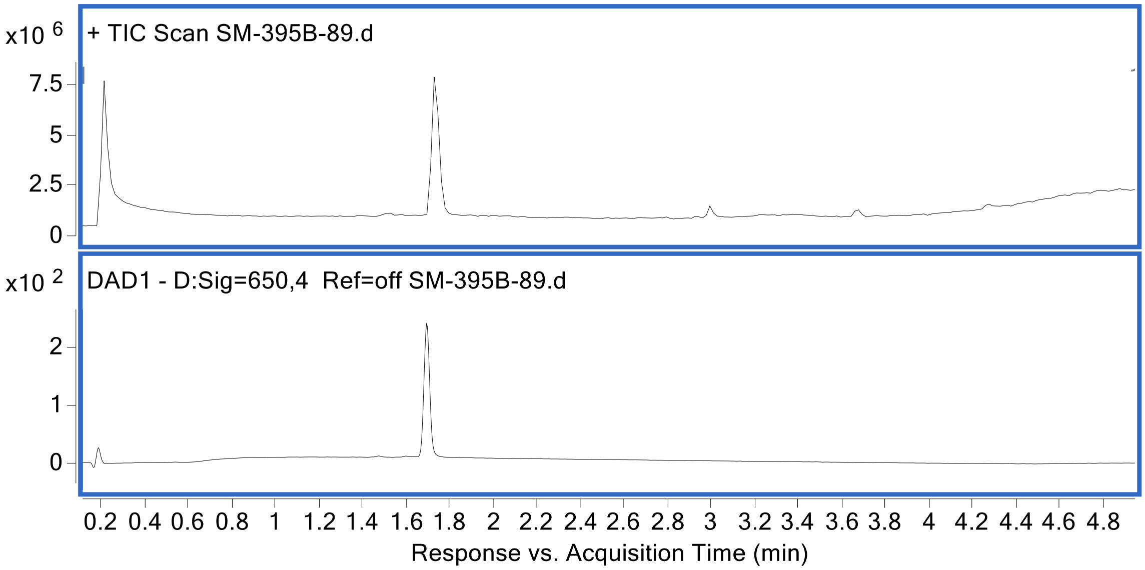
x10 6
+ TIC Scan SM-395B-89.d
7.5
5
2.5
0
x10 2
DAD1 - D:Sig=650,4 Ref=off SM-395B-89.d
2
1
0
0.2 0.4 0.6 0.8 1 1.2 1.4 1.6 1.8 2 2.2 2.4 2.6 2.8 3 3.2 3.4 3.6 3.8 4 4.2 4.4 4.6 4.8
Response vs. Acquisition Time (min)

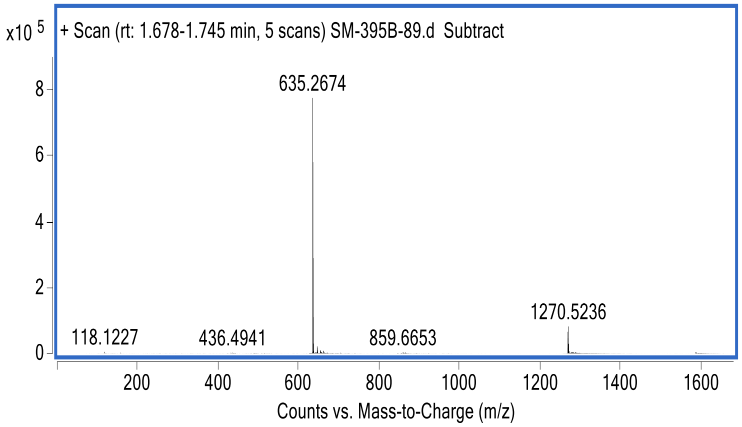
x10 5
+ Scan (rt: 1.678-1.745 min, 5 scans) SM-395B-89.d Subtract
635.2674
118.1227
436.4941
859.6653
1270.5236
8
6
4
2
0
200 400 600 800 1000 1200 1400 1600
Counts vs. Mass-to-Charge (m/z)

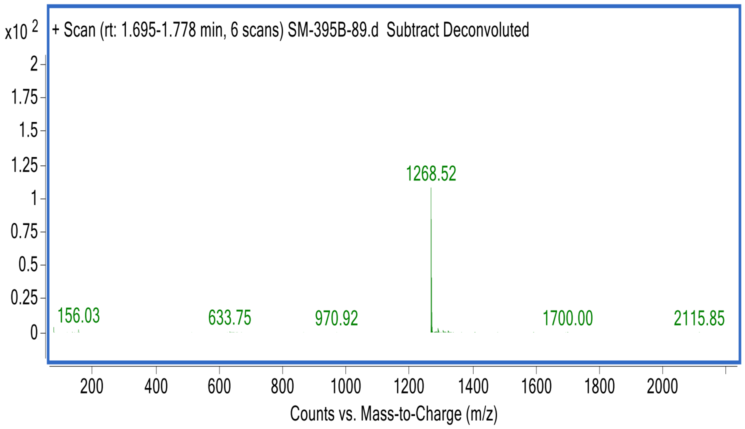
x10 2
+ Scan (rt: 1.695-1.778 min, 6 scans) SM-395B-89.d Subtract Deconvoluted
1268.52
156.03
633.75
970.92
1700.00
2115.85
2
1.75
1.5
1.25
1
0.75
0.5
0.25
0
200 400 600 800 1000 1200 1400 1600 1800 2000
Counts vs. Mass-to-Charge (m/z)

HRMS of **qABP 5**

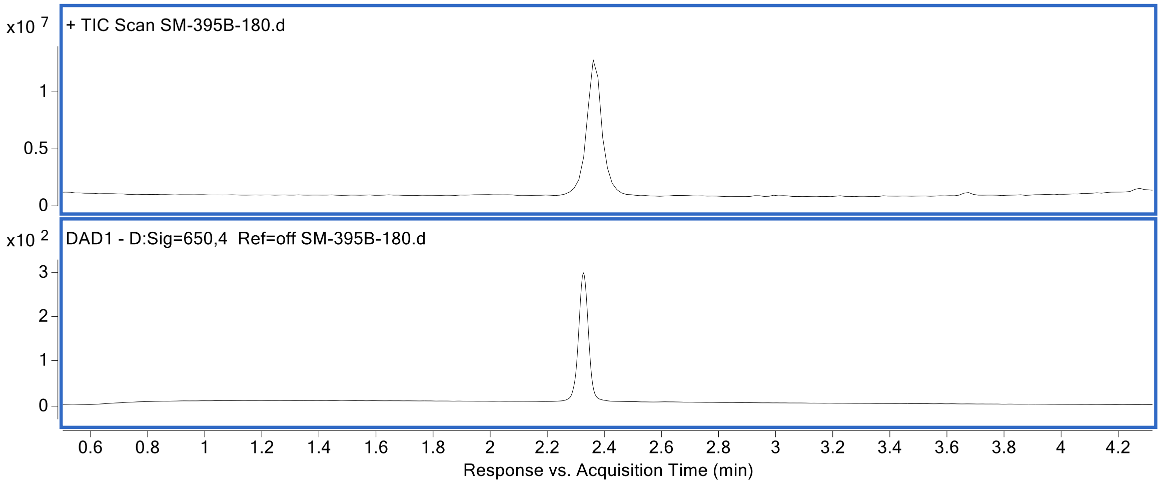

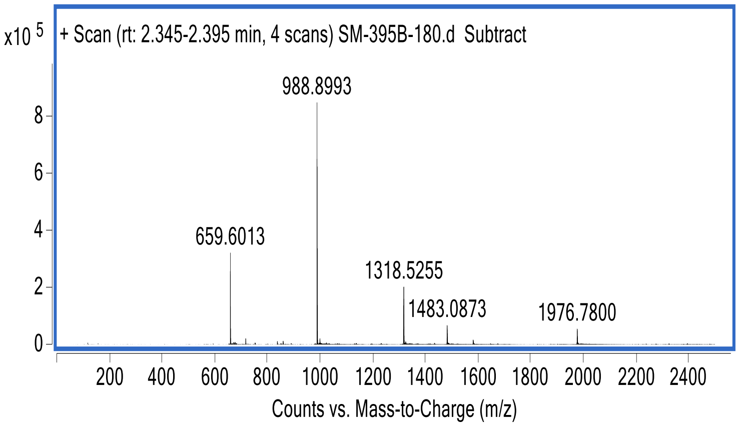

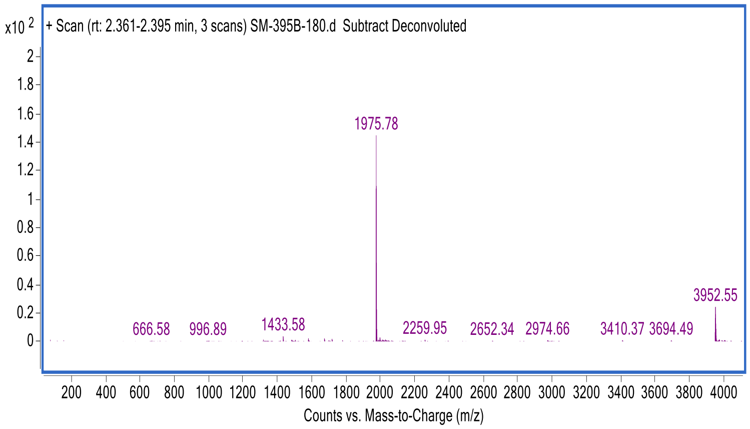

HRMS of **qABP 6**

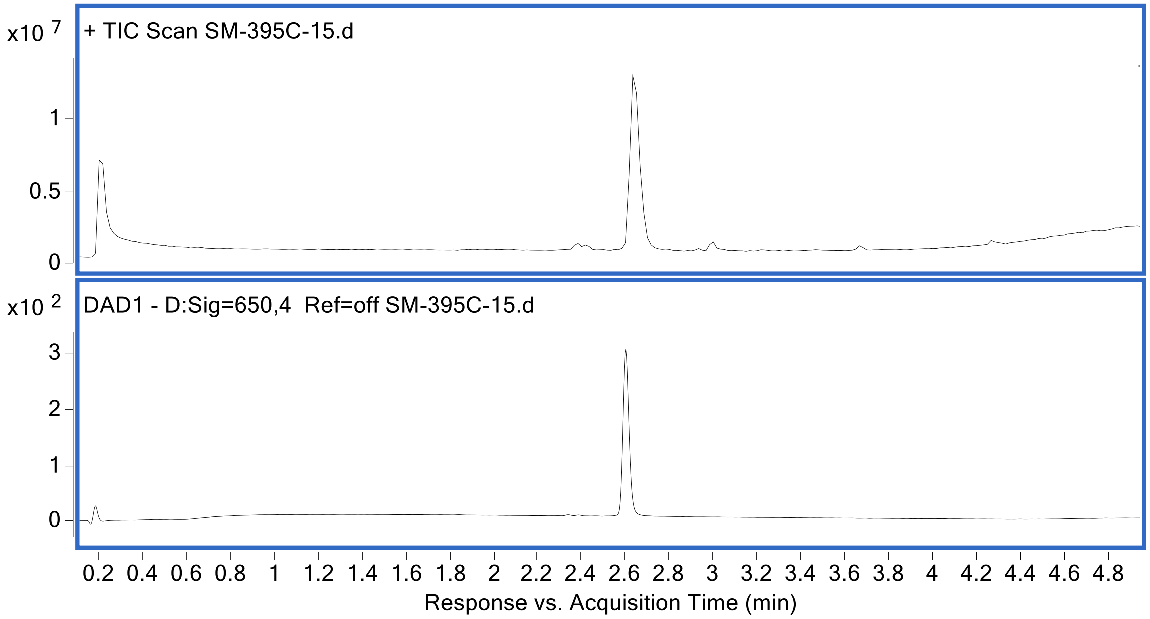

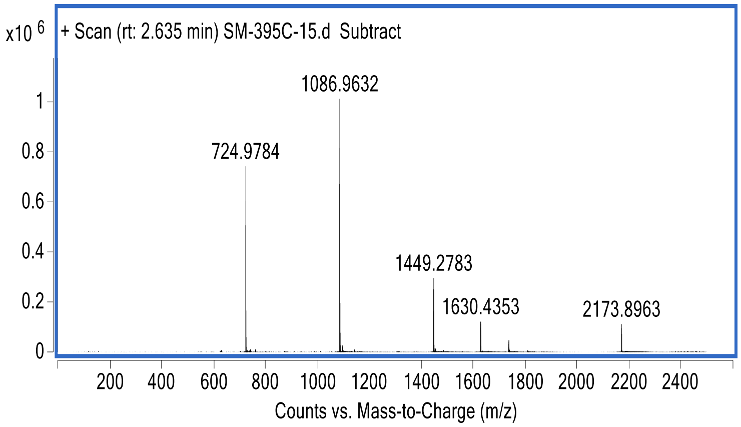

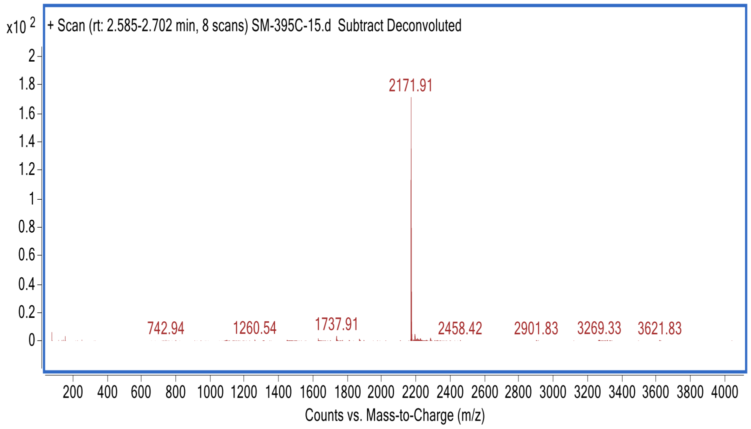

HRMS of **qABP 7**

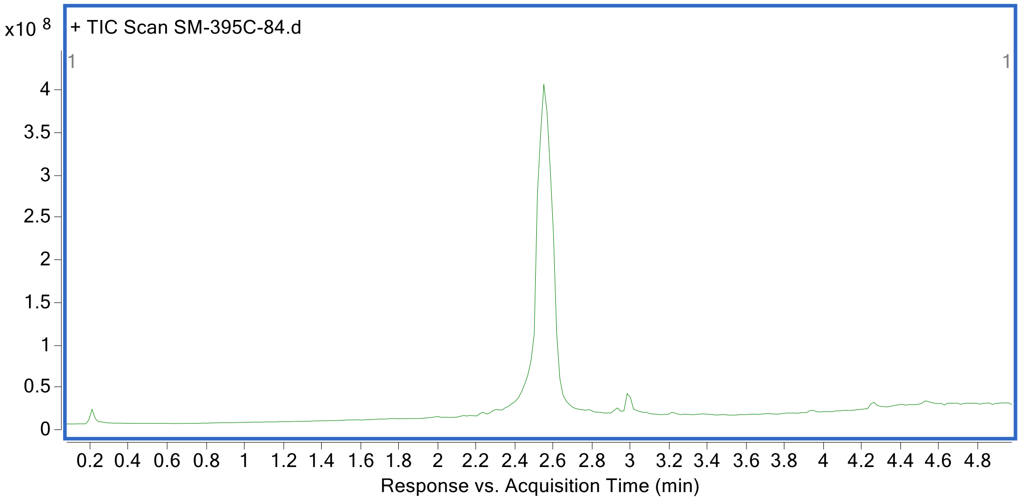

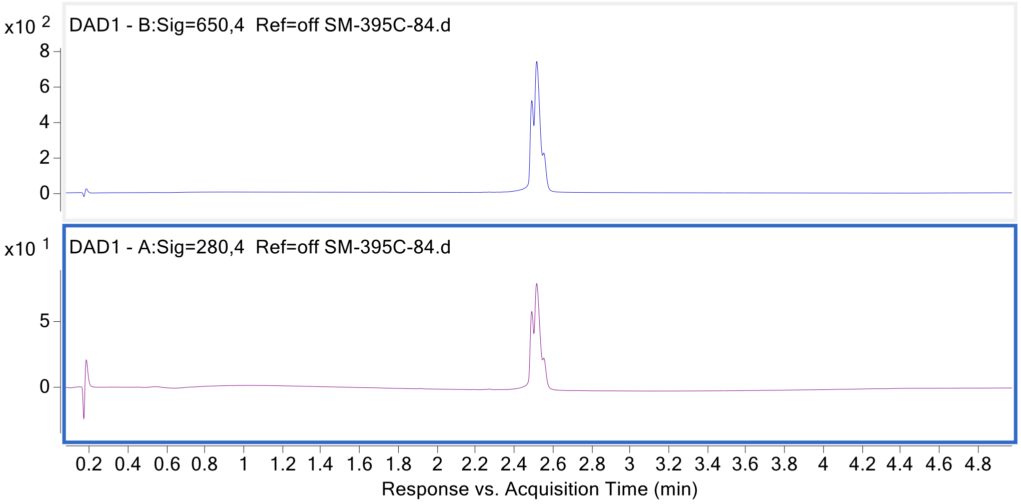

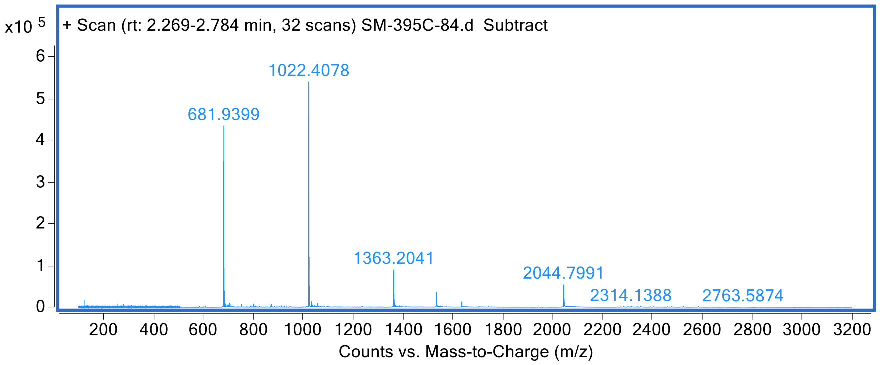

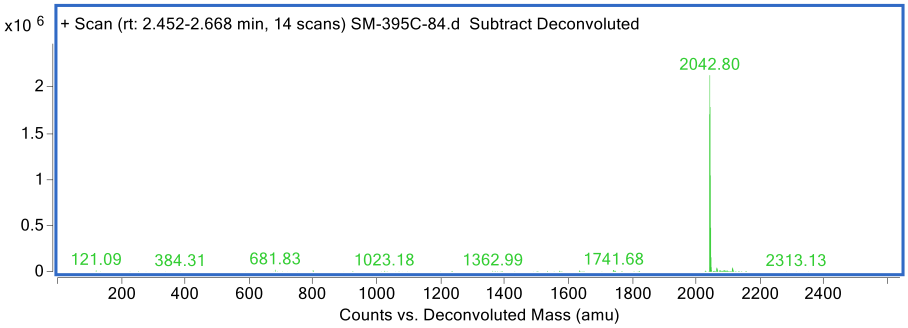
x10 6
+ Scan (rt: 2.452-2.668 min, 14 scans) SM-395C-84.d Subtract Deconvoluted
2042.80
121.09
384.31
681.83
1023.18
1362.99
1741.68
2313.13
Counts vs. Deconvoluted Mass (amu)

References

[1] S. Ji, S. H. L. Verhelst. Furin-targeting activity-based probes with phosphonate and phosphinate esters as warheads. *Org Biomol Chem* **2023**, *21*, 6498-6502.
[2] L. Zhang, S. Lovell, E. De Vita, P. K. A. Jagtap, D. Lucy, A. Goya Grocin, S. Kjaer, A. Borg, J. Hennig, A. K. Miller, E. W. Tate. A KLK6 Activity-Based Probe Reveals a Role for KLK6 Activity in Pancreatic Cancer Cell Invasion. *J Am Chem Soc* **2022**, *144*, 22493-22504.
